# Can Urban Grassland Plants Contribute to the Phytoremediation of Soils Contaminated with Heavy Metals

**DOI:** 10.3390/molecules27196558

**Published:** 2022-10-04

**Authors:** Zvjezdana Stančić, Željka Fiket, Dinko Vujević

**Affiliations:** 1 Faculty of Geotechnical Engineering, University of Zagreb, HR-42000 Varaždin, Croatia; 2 Division for Marine and Environmental Research, Ruđer Bošković Institute, HR-10000 Zagreb, Croatia

**Keywords:** phytoextraction, bioconcentration factor, *Taraxacum officinale*, *Plantago lanceolata*, *Trifolium repens*, biomass, unwashed and washed plant samples, Varaždin, Croatia

## Abstract

The main objective of this study was to investigate whether the most common wild plant species of urban grassland can be used for phytoremediation of soils polluted with heavy metals. The study was conducted in the city of Varaždin, in northern Croatia. The content of heavy metals (Cd, Cu, Fe, Mn, Ni, Pb, Zn) was determined in soil samples as well as in unwashed and washed plant samples (*Taraxacum officinale*, *Plantago lanceolata*, *Trifolium repens*). The results show that the most polluted site is the railway station, while most sites are polluted by road traffic. The soils are most enriched with Pb, Cu, Zn and Cd. The bioconcentration factors for all three plant species are <1, indicating the relatively low capacity of phytoextraction. A considerable amount of heavy metals is found in the dust deposited on the plant surface, which is confirmed by a statistically significant difference between washed and unwashed plant samples. In addition, the biomass of each plant species that can be removed (in t/ha year), the mass of specific heavy metal that can be removed (in kg/ha), and the years required for phytoremediation are reported. In conclusion, phytoremediation with only common plant species of urban grassland is not possible within a reasonable period of time.

## 1. Introduction

Heavy metal pollution of soil is widespread in urban areas. It is caused by emissions from road and rail transport, industry, fossil fuel combustion, waste disposal, corrosion and abrasion of materials, smelting of metals, mineral fertilizers, pesticides, flooding, etc. Numerous studies on the content of heavy metals (hereafter HMs) in urban soils have been carried out worldwide, including cities of the surrounding countries: in Austria [1], Bosnia and Herzegovina [2], Germany [3,4], Hungary [5,6], Italy [7,8], Serbia [9,10], Slovakia [11], Slovenia [12,13], Spain [14], and in many others. Since half of the world’s population lives in cities [15], intensive research is being conducted to find solutions to this problem. One of the main reasons for the great interest in this topic is the toxic effects of heavy metals on all living organisms, including humans [16,17,18].

In this context, phytoremediation has been established as an environmentally friendly technology for soil purification through natural processes using plants [19,20,21,22,23,24,25]. The most commonly studied form of phytoremediation is phytoextraction [21,23,25], in which plants take up pollutants from a polluted medium, usually soil, through their roots, and translocate them to aboveground biomass, which is then removed from the polluted area. The efficiency of phytoextraction is measured by the bioconcentration factor (BCF), which is the ratio of pollutant concentration in plant tissue to soil. Plant species whose BCF is >1 are considered good candidates for phytoextraction. Plants with extremely high BCFs are called hyperaccumulators [24,26,27] and constitute < 0.2% of angiosperms, and many of them are known so far from the *Brassicaceae* family [23]. Numerous studies are trying to find plant species that have the highest possible efficiency of phytoextraction [24,27,28,29]. However, other factors also play a role in evaluating the success of phytoextraction: biomass produced by plants, protection of nature and the environment, costs, etc.

Native wild plant species have great potential for phytoextraction [28]. Their advantage is that they do not pose a threat to the biodiversity, as their use does not require the introduction of invasive alien or genetically modified plant species that can negatively impact ecosystems. The green areas of cities consist mostly of grassland. Among the most common native wild plant species of urban grassland in temperate regions of Croatia and Europe are *Taraxacum officinale* agg. (dandelion), *Plantago lanceolata* L. (narrowleaf plantain), and *Trifolium repens* L. (white clover). Of the three species mentioned, the most data on heavy metal accumulation are found for *Taraxacum officinale* [30,31,32,33,34,35,36,37,38], while there are significantly fewer for *Plantago lanceolata* [13,31,39] and *Trifolium repens* [40,41,42]. Of the papers cited, relatively few relate to research on phytoremediation in urban areas [13,31,33,36,37]. Another problem is that biomass of plant species is rarely determined, and when it is, it is usually of cultivated species [21,43]. Furthermore, such studies rarely provide an estimate of the total mass of heavy metals that can be removed by mowing the aboveground parts of plant species over a given period of time. The deposition of dust and heavy metals on the surface of plants is also rarely considered; most studies have been conducted on woody species, which play a role in air purification in urban areas and serve as phytobarriers [44,45,46], and less frequently on herbaceous plants [42]. In Croatia, there are currently no studies on phytoremediation in urban areas, except for one study that preceded this work and included several species and a small number of sites in the city of Varaždin [47].

In this study, a number of methods were combined in order to obtain the best insight into the feasibility of phytoremediation. The main aim of this study was to investigate the possibility of phytoremediation of HMs (Cd, Cu, Fe, Mn, Ni, Pb and Zn) with common wild plant species (*Taraxacum officinale*, *Plantago lanceolata* and *Trifolium repens*) during regular mowing of urban grassland in the area of the city of Varaždin in northern Croatia. The specific objectives were: (i) to determine the degree of soil contamination with HMs (Cd, Cu, Fe, Mn, Ni, Pb, Zn), (ii) to determine the heavy metal content in the aboveground parts of *Taraxacum officinale*, *Plantago lanceolata* and *Trifolium repens*, (iii) to estimate the proportion of the mentioned plant species in the total biomass of urban grassland, (iv) to estimate the total mass of HMs that can be removed by mowing the urban grassland per unit area and cleanup time, and (v) to determine the difference in heavy metal content between washed and unwashed plant samples.

The main conclusions of this study are: (i) soils in the Varaždin city range from uncontaminated to heavily contaminated with heavy metals, (ii) the studied wild plant species (*Taraxacum officinale*, *Plantago lanceolata* and *Trifolium repens*) accumulate relatively low amounts of heavy metals in the aboveground parts (BCFs < 1), and (iii) although they constitute about 40% of the total dry biomass of urban grassland and do not require seeding or special care, phytoremediation of polluted sites based on these plant species alone would take from several decades (Zn, Cd, Cu) to several thousands (Pb).

## 2. Results and Discussion

### 2.1. Soil Properties

Soil properties (organic matter content, carbon content, pH_H2O_, pH_KCl_, and grain size fractions) and total concentrations of HMs (Cd, Cu, Fe, Ni, Mn, Pb, and Zn) in soil are presented as descriptive statistics in Table 1, all data in Table S1, and site descriptions in Table S2.

According to the soil reaction, the pH_H2O_ and pH_KCl_ of soil samples range from 7.18 to 8.49 and from 6.35 to 7.42, respectively. Most or 13 samples are slightly alkaline (7.6–8.1), one sample is very slightly alkaline (7.1–7.5) and two samples are moderately alkaline (8.2–8.6). Literature confirms that many urban soils are alkaline [5,6,7]. Puskás and Farsang [6] attribute this to debris, NaCl and CaCl_2_ used for deicing roads, which tend to increase the pH of the local soil. Since HMs are absorbed and thus immobilized in alkaline soils [48], this property can be considered beneficial because fewer HMs are available to living organisms.

Depending on their texture, the studied soils were classified into the following classes (Table S1): sandy loam as the most common (11 samples), followed by silty loam (four samples) and loamy sand (one sample). Among soil particles, coarse sand (2.0–0.2 mm) is the most common, with an average of 31.4%, followed by fine silt (0.02–0.002 mm) with an average of 23.5%, fine sand (0.2–0.063 mm) with an average of 21%, coarse silt (0.063–0.02 mm) with average of 20.1%, and clay (> 0.002 mm) with an average of 3.96%. The particle size of the soil can significantly affect the accumulation of HMs, so that the content of most HMs is higher in soils with fine particles than in soils with coarse particles [49]. Biasioli et al. [7] consider that urban soils usually contain coarser fractions due to the admixture of foreign material.

The organic matter in samples varied from 1.63 to 8.66% with an average of 4.88%. Of the 16 samples, six samples belong to very humus-rich soils (class range: 5.1–10%), eight samples belong to fairly humus-rich soils (3.1–5%), one sample belongs to medium humus-rich soils (2.1–3%), and one sample belongs to low humus-rich soils (1.1–2.0%). The high content of organic matter can also be considered as a favorable characteristic, since it causes a high sorption capacity for HMs and thus their immobilization [50]. In this way, the organic matter reduces the bioavailability of HMs and has a protective function.

### 2.2. Heavy Metals in the Soil

#### 2.2.1. Distribution of HM in Soils

As shown in Table 1, the soils studied have a wide range of HM values. The highest values for six of the seven HMs were determined at the Varaždin railway station (site 13, Table S1): for Cd (2.12 mg/kg), Cu (161 mg/kg), Fe (52.0 g/kg), Ni (73.1 mg/kg), Pb (490 mg/kg), and for Zn (481 mg/kg), indicating that this is the most polluted site. Only for Mn was the highest value (973 mg/kg) measured along the busy road (site 3, Table S1). The second most polluted site is Drava Island, which is affected by flooding of the Drava River (site 1, Table S1). These two sites can be considered as pollution hotspots. They are followed by the areas under the influence of road traffic, which make up most of the city of Varaždin, and then the areas under the synergistic influence of industry and road traffic.

Compared to other European cities [1,3,4,5,6,7,9,10,11,12,13], the soils of the city of Varaždin had the highest concentrations of Fe and Mn, while Cd, Cu, Ni, Pb, and Zn were found in lower concentrations (Table S3).

It is known that the soils of urban areas are enriched in HMs compared to natural background levels. The upper soil layers are more contaminated than the deeper ones [5,51]. There are also significant differences between the different functional sites. The most polluted areas are located within or near the city centers [3,10,13]. In some works [1,5,10] road traffic is mentioned as the main source of pollution, but the authors do not exclude other sources. Regarding road traffic, the levels of HMs are highest near roads and decrease with increasing distance [2,11,51] and pollution is positively correlated with average daily traffic frequency (cars/day) [11]. Charleswort et al. [52] and Hiller et al. [11] pointed out the enrichment of soil with Sb, Cu, Pb and Zn near traffic intersections with traffic lights where vehicles slow down. According to Hjortenkrans et al. [51], the distribution of HMs is also influenced by the design of road construction and roadside terrain, as they determine the runoff paths of stormwater. Birke and Rauch [4] in Berlin, Norra et al. [3] in Pforzheim (Germany) and Leštan et al. [13] in Celje (Slovenia) cite old industrial areas as the most polluted. This is a historical soil contamination accumulated over many years, showing the persistence of HMs as pollutants. Some authors also found that areas along railway lines are heavily polluted with HMs, which is also the case in the study area of Varaždin. Staszewski et al. [53] pointed out that the concentrations of HMs in soils near investigated railway lines and railway junctions in Poland are higher than along traffic roads and in urban centers. Wan et al. [54] studied HMs in dust in various functional areas in the northern Chinese city of Shijiazhuang and found that the area around the North Railway Station was among the most polluted. Accordingly, railway stations and railway junctions in urban areas, often located near city centers, are places with very high pollution levels that should not be neglected.

#### 2.2.2. Soil Pollution Level

In Croatia, the level of soil contamination has been set according to the Ordinance on the Protection of Agricultural Land from Pollution [55], and the maximum permissible levels depend on the soil reaction. A comparison of total concentrations of HMs with the above-mentioned regulations of the Republic of Croatia [55] (Table S4) shows that the maximum permissible concentrations are exceeded for Pb (four sites), Zn (two sites), Cd (one site) and Cu (one site). A comparison with the regulations of the Netherlands (Table S4) shows that in the city of Varaždin no value exceeds the intervention values [56], while the target values [57] are exceeded for Cu (10 sites), Ni (nine sites), Pb (eight sites), Zn (four sites) and for Cd (three sites).

Relative to the mean values of HMs in Croatia [58], contamination factors (CFs) for 16 investigated sites (Table S5a) showed the highest Pb load (at one site CF = 12.9 and at 15 sites CFs > 1), followed by Cu (at one site CF = 5.4 and at 14 sites CFs > 1), and then Zn (at 11 sites CFs > 1), while for Cd, Fe, Mn and Ni at three, three, two and two sites, respectively, CFs > 1. Similar results were obtained when CFs were calculated relative to the mean values of the studied HMs for Europe [59] (Table S5b), with Pb having the highest values (at one site CF = 15, at one site CF = 6.3 and at 15 sites CFs > 1), followed by Cu (at one site CF = 9.3 and at all 16 sites CFs > 1), then Zn (at two sites CFs > 5 and at 15 sites CFs > 1), and then Cd (at one site CF = 7.6 and at 11 sites CFs > 1), while Ni, Fe, and Mn had CFs > 1 at seven, three, and two sites, respectively. Considering the average CF values of the specific HMs calculated in relation to the mean values for Europe, they are ordered as follows: Pb > Cu > Zn > Cd > Ni > Fe > Mn.

Looking at individual sites based on calculated CFs (Table S5a, b), Varaždin railway station (site 13) is the most polluted, followed by Drava Island (site 1), Koprivnička Street (site 12) and Svilarska Street (site 14). All other sites along roads, intersections, parking lots, industrial areas, gas stations, parks and waste bales had lower CF values.

**Figure 1:**
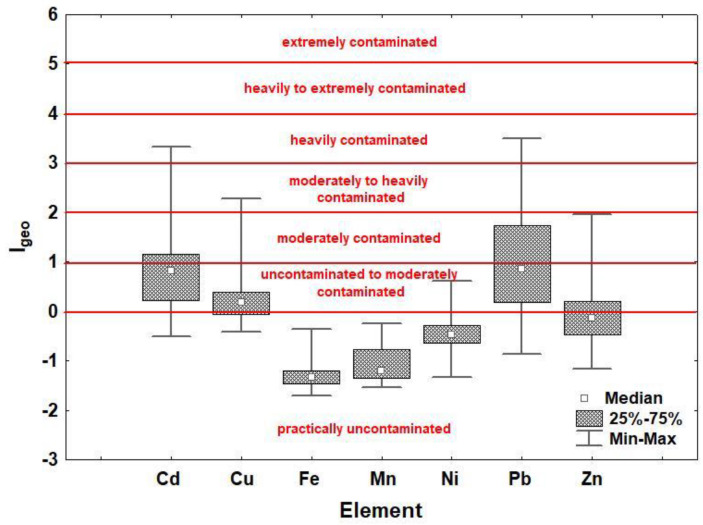
Geoaccumulation indices (I_geo_) for the studied heavy metals (Cd, Cu, Fe, Mn, Ni, Pb and Zn) calculated in relation to the mean values of heavy metals in the region [59] with reported pollution levels [60] for soils in the area of Varaždin (N Croatia).

The geoaccumulation indices (I_geo_) calculated in relation to the mean values of HMs in Croatia [58], Europe [59] and the region [59] are shown in Table S6. The highest contamination levels [60] were obtained by calculating I_geo_ in relation to the mean values of HMs for the region (Table S6c), which is also shown in Figure 1. According to this, all the studied soils can be considered practically uncontaminated for Fe and Mn (I_geo_ ≤ 0). The same is true for Ni, except for two sites where the soils are uncontaminated to moderately contaminated (0 < I_geo_ < 1). For Cu and Zn, the highest level of contamination is found at one site and belongs to moderately to heavily contaminated soils (2 < I_geo_ < 3), while for Cd and Pb, one site can even be considered heavily contaminated (3 < I_geo_ < 4).

The average values of I_geo_ correspond to the order of CFs: Pb > Cu > Zn > Cd > Ni > Fe > Mn (both calculated in relation to the mean values of HMs for Europe).

Considering the I_geo_ of individual sites, the most contaminated are Varaždin railway station (site 13), Drava Island (site 1), Svilarska Street (site 14) and Koprivnička Street (site 12). The number of completely uncontaminated sites differs according to the I_geo_ values calculated for Croatia, Europe and the region, where six, two, and one sites were identified, respectively.

#### 2.2.3. Influence of Soil Properties on HM Level

In addition to the total concentrations of heavy metals in the soil, other physical and chemical properties of the soil can affect the solubility, mobility, and bioavailability of heavy metals. Therefore, the influence of soil reaction, soil texture and organic matter content on HMs level in soils was investigated.

The correlation analysis (Table S7) showed a statistically significant positive correlation between most of the heavy metals studied (Cd, Cu, Fe, Ni, Pb, and Zn), indicating, at least in part, their common source, with the exception of Mn. The percentage of organic matter or organic carbon showed a statistically significant correlation with the Cd and Pb content in the soil, implying that both elements are adsorbed to a significant extent on organic matter, while the soil reaction and soil fractions showed no correlation with the heavy metals. Unfortunately, a more detailed analysis of the measured parameters and their correlations is prevented by a relatively small data set.

### 2.3. Heavy Metals in Plants

#### 2.3.1. Total Concentrations of Heavy Metals in Washed Plant Samples

Total HM concentrations in washed samples of the three most common plant species of urban grassland, *Taraxacum officinale* (dandelion), *Plantago lanceolata* (narrowleaf plantain) and *Trifolium repens* (white clover), are presented as descriptive statistics in Table 2, while all data are shown in Table S1.

Of the heavy metals studied, *Taraxacum officinale* has the highest mean accumulation values for Cd (0.38 mg/kg), Mn (34 mg/kg), and Ni (1.19 mg/kg), *Plantago lanceolata* for Fe (139 mg/kg), Pb (0.93 mg/kg), and Zn (104 mg/kg), and *Trifolium repens* only for Cu (17.4 mg/kg) (Table 2).

A comparison of mean concentrations of heavy metals in the aboveground parts of *Taraxacum officinale* in different parts of Europe [30,31,32,33,34,35,36,37,38] (Table S8) (N—number of samples = 1037, 1040, 947, 972, 994, 1048, 1052; Cd—0.59 mg/kg, Cu—10.6 mg/kg, Fe—235 mg/kg, Mn—57.8 mg/kg, Ni—3.3 mg/kg, Pb—2.5 mg/kg, Zn—46.8 mg/kg) with those in the area of Varaždin (N = 16, 16, 16, 16, 15, 16, 16; Cd—0.38 mg/kg, Cu—15.1 mg/kg, Fe—125 mg/kg, Mn—33.7 mg/kg, Ni—1.19 mg/kg, Pb—0.24 mg/kg, Zn—92.1 mg/kg) shows that the values for Cd, Fe, Mn, Ni and Pb are higher in the area of Europe, and for Cu and Zn in the area of Varaždin.

Comparison of mean concentrations of heavy metals in the aboveground parts of *Plantago lanceolata* in different parts of Europe [13,31,39] (Table S8) (N = 23, 23, 10, 23, 0, 48, 48; Cd—0.19 mg /kg, Cu—8.8 mg/kg, Fe—11.5 mg/kg, Mn—19.7 mg/kg, Pb—2.57 mg/kg, Zn—98 mg/kg) with those in the Varaždin area (N = 15, 14, 15, 15, 15, 15, 14; Cd—0.25 mg/kg, Cu—9.83 mg/kg, Fe—139 mg/kg, Mn—27.4 mg/kg, Ni—0.95 mg/kg, Pb—0.93 mg/kg, Zn—104 mg/kg) shows that the values are higher only for Pb in Europe and for Cd, Cu, Fe, Mn, and Zn in the Varaždin area, while for Ni, a comparison is not possible due to the lack of data.

Comparison of mean concentrations of heavy metals in the aboveground parts of *Trifolium repens* in different parts of Europe [40,41,42] (Table S8) (N = 15, 3, 0, 0, 3, 15, 15; Cd—6.41 mg/kg, Cu—16.46 mg/kg, Ni—88.2 mg/kg, Pb—29.1 mg/kg, Zn—92 mg/kg) with those in the Varaždin area (N = 15, 15, 15, 15, 15, 15, 14; Cd—0.03 mg/kg, Cu—17.4 mg/kg, Fe—138 mg/kg, Mn—24.7 mg/kg, Ni—0.83 mg/kg, Pb—0.52 mg/kg, Zn—56.7 mg/kg) shows that the values for Cd, Ni, Pb and Zn are higher in the area of Europe and for Cu in the area of Varaždin, while for Fe and Mn, a comparison is not possible due to the lack of data.

Although all three species studied are very common in the Croatian and European flora, only for *Taraxacum officinale* are there extensive data on HMs in the aboveground parts, while the data for *Plantago lanceolata* and *Trifolium repens* are rather sparse (Table S8). Most of the mean concentrations compared in Table S8 are of the same order of magnitude, while where there are few data there are deviations of one or two orders of magnitude. Variations in HM values are likely in part the result of different protocols for sample preparation and analysis.

The accumulation of essential HMs depends partly on the needs of plant species. According to Kirkby [61] the average concentrations of micronutrients in the dry matter of plant shoots sufficient for adequate growth are as follows: Cu—6 mg/kg, Fe—100 mg/kg, Mn—50 mg/kg, Ni—0.1 mg/kg, and Zn—20 mg/kg. Comparison of the above values with the mean values of HMs in the three studied plant species shows that in the Varaždin area the values for Cu, Fe, Ni and Zn are exceeded, and there is a deficiency only for Mn.

The determined mean values of HMs in the studied plant species in the Varaždin area were also compared with the element contents in plants in general. For example, Markert [62] gives the following average contents in plants (in dry weight): Cd—0.03–0.5 mg/kg, Cu—2–20 mg/kg, Fe—5–200 mg/kg, Mn—1–700 mg/kg, Ni—0.4–4 mg/kg, Pb—0.1–5 mg/kg, and Zn—15–150 mg/kg. The comparison showed that the average values for all HMs and all three studied plant species are within the range given by Markert [62]. In addition, Kabata-Pendias and Pendias [63] give approximate sufficient or normal concentrations of chemical elements in leaf tissues expressed in dry weight generally for different plant species as follows: Cd—0.05–0.2 mg/kg, Cu—5–30 mg/kg, Mn—30–300 mg/kg, Ni—0.1–5 mg/kg, Pb—5–10 mg/kg, and Zn—27–150 mg/kg. The comparison shows that for Cu, Ni and Zn, all average values from the Varaždin area are within the indicated ranges [63]; for Cd in *Taraxacum officinale* and *Plantago lanceolata*, the values are above and for *Trifolium repens* below the indicated range; for Mn in *Plantago lanceolata* and *Trifolium repens*, the values are below the range, similar to the comparison with the values according to Kirkby [61]; interestingly, for Pb, all average values are below the indicated ranges, while for Fe, a comparison is not possible due to the lack of data.

**Figure 2:**
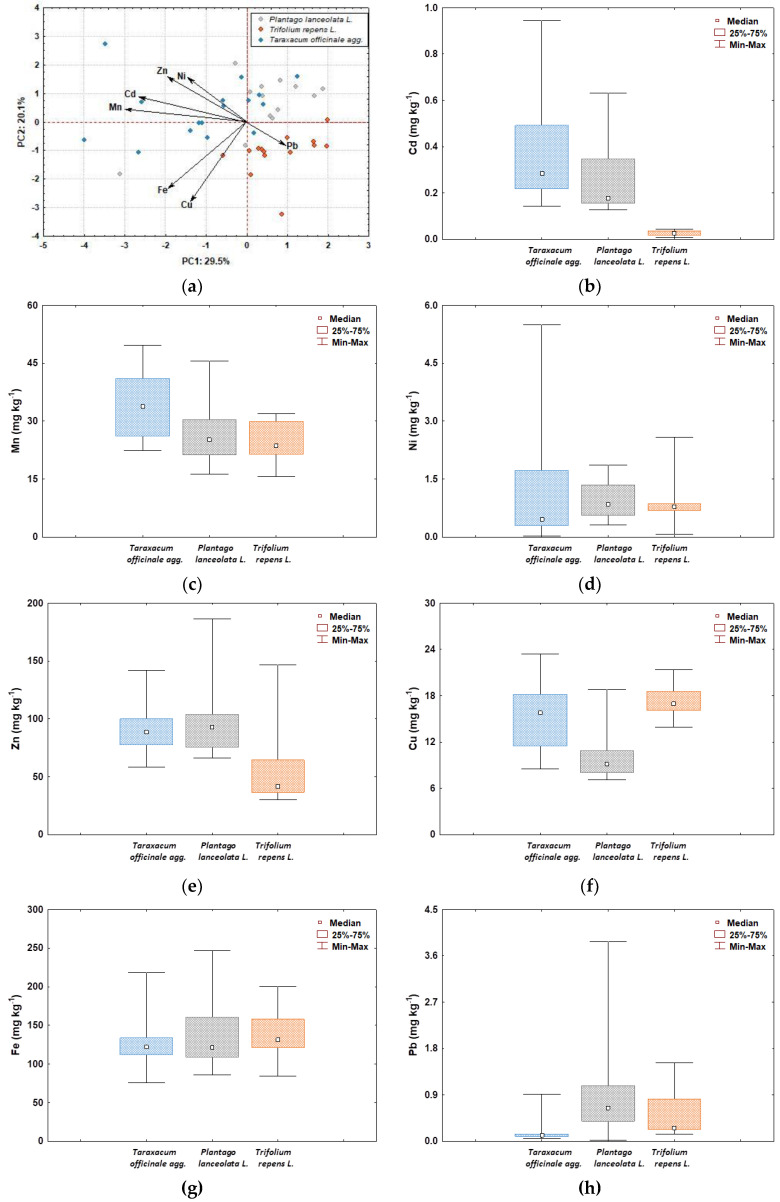
PCA biplot (**a**) and boxplots of total HM concentrations (Cd, Cu, Fe, Mn, Ni, Pb, and Zn) in washed samples of *Taraxacum officinale*, *Plantago lanceolata*, and *Trifolium repens* **(b**–**h)**.

The PCA and boxplot analyses of heavy metal content in the studied plant species from the Varaždin area show different patterns in terms of values and ranges (Figure 2). As can be seen from the PC1 vs. PC2 plot (Figure 2a), three groups of HMs are separated. The first group includes Cd, Mn, Ni, and Zn, and is characterized by their higher accumulation in *Taraxacum officinale* and *Plantago lanceolata* than in *Trifolium repens* (Figure 2b–e). The second group consists of Cu and Fe, which are accumulated to a considerable extent in *Trifolium repens* and *Taraxacum officinale* and to a slightly lesser extent in *Plantago lanceolata* (Figure 2f,g). The Pb is separated in the third group from the other elements, being more enriched in *Plantago lanceolata* and *Trifolium repens* and less in *Taraxacum officinale* (Figure 2h).

#### 2.3.2. Factors Affecting Phytoextraction of Heavy Metals

**Figure 3:**
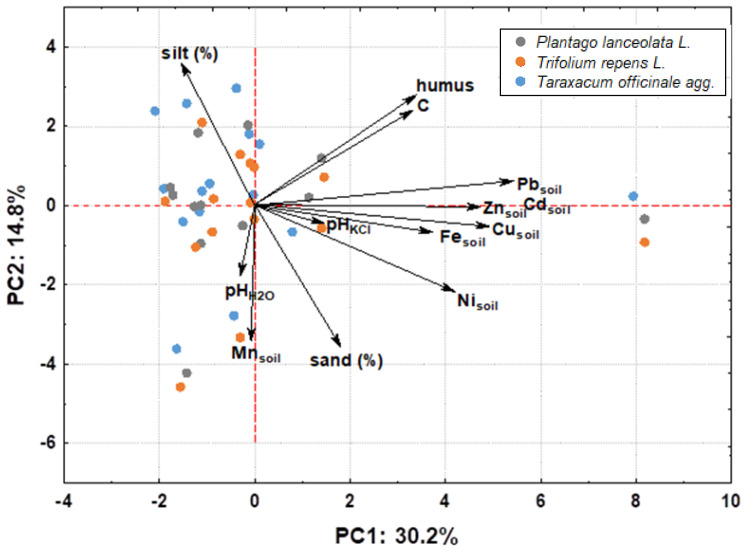
PCA plot showing concentrations of heavy metals in washed plant samples (*Taraxacum officinale*, *Plantago lanceolata*, and *Trifolium repens*) in relation to other parameters studied, such as concentrations of heavy metals in soil, texture, soil pH, and organic matter.

In examining the factors affecting phytoextraction, PCA analysis (Figure 3) further revealed that the HM content in the plants studied does not depend simply on the accumulation ability of the species, but rather on multiple factors, soil texture (represented here by silt content), organic matter content (humus), and geochemical composition, i.e., HM content in the soil.

Correlation analyses (Table S9) showed that the Fe content in plants is influenced by the humus content and the concentration of Pb, Cu, Fe, and Ni in the soil, and there is also a positive correlation between the Fe and Cu content in plants, probably indicating a similar uptake mechanism. The Pb content in plants was influenced by the concentrations of Pb, Fe, Cd, Zn, and Cu in the soil, as shown by statistically significant correlations (Table S9). Mn content in plants, on the other hand, was associated with Ni and Cd content in plants, while Zn content was associated with Cd content in plants (Table S9). In this context, Rascio and Navari-Izzo [27] explain the connection between Zn and Cd uptake with the presence of specialized protein transporters in the plasma membrane of root cells that enable the influx of these elements. Consequently, from the correlation analyses, it can be concluded that the composition of Fe and Pb in *Taraxacum officinale*, *Plantago lanceolata*, and *Trifolium repens* is influenced by soil composition, while Mn and Zn are influenced by mechanisms of uptake or bioavailable forms.

In scientific works investigating the influence of various factors on the concentration of HMs in the aboveground parts of *Taraxacum officinale*, *Plantago lanceolata* and *Trifolium repens*, different results were obtained. Kabata-Pendias and Dudka [35] and Kabata-Pendias and Krakowiak [36] concluded that specimens of *Taraxacum officinale* growing in areas with higher air and soil pollution with Cd, Pb and other HMs, also have higher concentrations of these elements in the leaves. Diatta et al. [37] determined a significant correlation between the content of HMs in the soil and the leaves of *Taraxacum officinale* only for Pb (among other HMs: Zn, Cu, Cd, and Ni). Bini et al. [32] note that *Taraxacum officinale* is able to accumulate HMs (Cu, Fe, Pb, Zn) in shoots at contaminated sites in much higher concentrations than at non-contaminated sites. Malizia et al. [33] found a linear dependence between Cu concentrations in soil and leaves of *Taraxacum officinale*. Leštan et al. [13] found for *Plantago lanceolata* that the uptake of Pb is not influenced by the total Pb content in the soil, Pb fractionation, or soil properties, which can be explained by a very low bioavailable Pb fraction in the soil; the uptake of Zn is influenced by the fraction of Zn soluble in the soil solution and the fraction of Zn exchangeable from soil colloids into the soil solution and increases with a decrease in soil pH. Bidar et al. [41] found for *Trifolium repens* that Cd, Pb, and Zn concentrations were higher in plants growing on contaminated soils than in the same plant species growing on uncontaminated soils.

The relationships between concentrations of HMs in plants have rarely been studied. Interestingly, Djingova and Kuleff [31] determined positive correlations between certain HMs in plants (*Taraxacum officinale* and *Plantago lanceolata*), but not for the same pairs as in this work.

In some studies, scientists went a step further and found that elevated HM concentrations in the soil reduced some morphological and anatomical characteristics as well as total biomass, as in the case of *Taraxacum officinale* growing on mine tailings [32].

The uptake of HMs in different plant species generally depends on a whole range of factors, which can be divided into edaphic and climatic factors and plant species characteristics.

Edaphic factors include: the total amount and bioavailable fractions of HMs in the soil, the complex relationships among HMs in the soil that may affect bioavailability, mineral composition, soil pH (many metal cations such as Cd, Cu, Hg, Pb, and Zn are reported to be present at low pH, i.e., below 5.5), soil particle size, soil colloids, soil aeration, soil moisture content and water holding capacity, soil organic matter content, soil sorption capacity, cation exchange capacity, and microbial activity [22,23,25].

While all these factors influence the behavior of metals in soils [64], soil pH is one of the most important parameters affecting the mobility of metals [65]. For most heavy metals, mobility generally increases at lower pH and decreases as soil pH increases. According to Kabata-Pendias and Pendias [63], cation mobility in the aqueous phase of soil may decrease under the oxidation regime in the following order: Mn^2+^ > Cd^2+^ > Ni^2+^ > Pb^2+^ > Zn^2+^ = Cu^2+^ > Fe^3+^, although this order may change somewhat depending on the soil properties and the depth of observation [65,66].

Climate also has a very important influence, especially temperature and precipitation [23].

Each plant species has a characteristic: pattern of HM uptake, tolerance to elevated HM concentrations in soil (chelation and detoxification/sequestration of metals by certain ligands are important mechanisms by which plants cope with HM-induced oxidative stress), ability to stimulate metal bioavailability in the rhizosphere, symbiotic organisms living associated with plants (bacteria, mycorrhizal fungi), genotypes within species, variation in HM uptake during the growing season, etc. [19,23,25,27,31,41,42].

#### 2.3.3. Bioconcentration Factors (BCFs)

**Figure 4:**
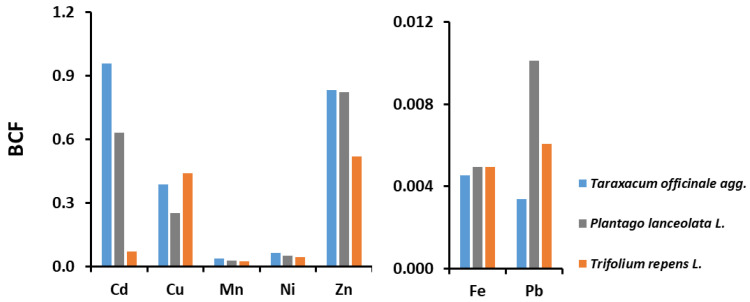
Bioconcentration factors (BCFs) for heavy metals (Cd, Cu, Fe, Mn, Ni, Pb and Zn) in three plant species (*Taraxacum officinale*, *Plantago lanceolata* and *Trifolium repens*) collected in the Varaždin area.

For the Varaždin area, the bioconcentration factors for all three plant species studied and for all seven HMs are < 1 (Figure 4) and therefore these plants cannot be considered hyperaccumulators [23,26,27]. The studied plant species showed the highest efficiency in removing Cd, Zn and Cu from the soil (the only exception is *Trifolium repens* for Cd), and the lowest for Fe and Pb (Figure 4). A comparison of the values of BCF from the Varaždin area with other studies with the same plant species in Europe [13,32,33,34,37,40,42,67] (Table S10) shows the same trend, i.e., most values are in the same order of magnitude, and only a few show differences of one or two orders of magnitude. The detail that should be highlighted is that other authors have obtained BCF values > 1 [32] and some even > 2 [37] for Cd in *Taraxacum officinale*, indicating that this species has the potential to remove this non-essential and toxic element.

#### 2.3.4. Differences in Heavy Metal Content between Unwashed and Washed Plant Samples

The mass concentrations of HMs in the dust deposited on the surface of the plant species are shown in Table 3. Each plant species has a specific pattern of dust deposition and associated content of HMs. The highest mass of Cd, Cu, Fe, Mn and Ni was found on the surface of *Trifolium repens*, Pb on *Plantago lanceolata* and Zn on *Taraxacum officinale*. HMs found on the surface of all three plant species were ordered by mass from largest to smallest: Fe > Zn > Mn > Cu > Pb > Ni > Cd. This order is very similar to the order of HMs by average mass concentrations in soil (Table 1) from highest to lowest values as follows: Fe > Mn > Zn > Pb > Cu > Ni > Cd.

A statistically significant difference was found between washed and unwashed plants (*p* < 0.05) (Table S11): at *Taraxacum officinale* for Fe, Pb, and Zn; at *Plantago lanceolata* for Cu, Fe, Mn, Pb, and Zn; and at *Trifolium repens* for Cd, Cu, Fe, Mn, and Pb.

**Figure 5:**
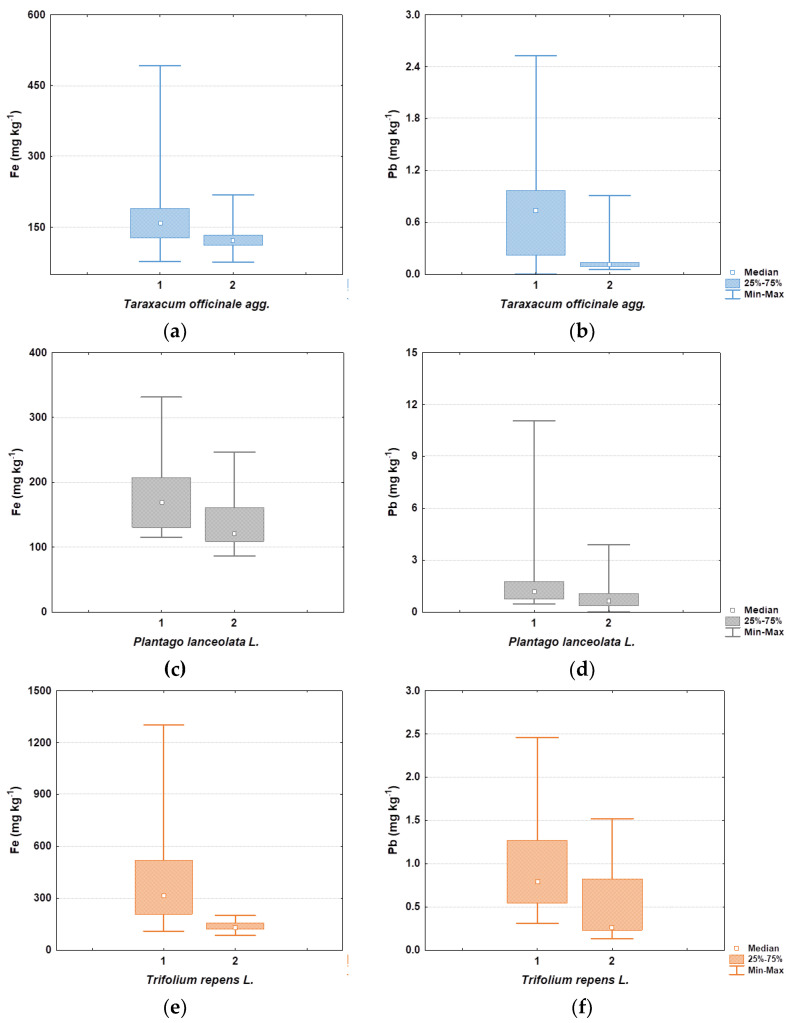
Boxplots showing differences in Fe and Pb content between unwashed (1) and washed (2) plant samples: (**a**,**b**) for *Taraxacum offiicinale*, (**c**,**d**) for *Plantago lanceolata*, and (**e**,**f**) for *Trifolium repens*.

From the results (Table 3), it can be concluded that *Trifolium repens* retains the largest amount of dust particles on the surface of the aboveground parts. It is also interesting to note that for all three species there was a statistically significant difference between the unwashed and washed plant samples for Fe and Pb, as shown in Figure 5.

Comparison with data from the literature is possible only for *Trifolium repens* and Cd, Pb, and Zn in the work of Bidar et al. [42], where the concentrations in unwashed shoots were significantly higher than in washed shoots, and the differences ranged from 3 to 68%.

According to Sánchez-López et al. [45], the retention of dust particles is enabled by rough leaf surfaces and different leaf structures such as trichomes, vines, glands, stomata and even fungal mycelium. In this study, however, it is clear that, besides the microstructures on the leaf surface that can serve as traps for dust particles, the spatial distribution and density of aboveground plant parts also play an important role. The reason why *Trifolium repens* retains most of the dust on its surface can be explained by the fact that its trifoliate leaves (without trichomes on the upper surface) are very numerous on the creeping stems and thus have a large surface area; moreover, its leaves have an almost horizontal position during the day, which allows dust to settle on their surface. In contrast, *Plantago lanceolata* and *Taraxacum officinale* have hairs on the upper surface of their leaves, but their leaves are more or less erect and, therefore, less dust can be deposited on their surface.

Determination of dust or particulate matter on the surface of plants is important for several reasons. First, it is an indicator of the pollution level of the air with particulate matter [68]. Second, it is an indicator of how efficient a certain plant species is in removing airborne dust [44,45,46], which is of practical value in urban or other areas with high level of an air pollution, for the removal of which woody species are usually used. Third, the retention of dust particles on the surface of plants should be considered in scientific research such as phytoremediation. As far as methodology is concerned, there are very different approaches. In some studies, even unwashed samples were used. However, washed samples are most commonly used, sometimes with clear water only, sometimes with distilled or deionized water only, sometimes with distilled water first and then with deionized water. Some works also use additional washing procedures with P-free detergent and immersion in dilute HCl [45]. However, detailed studies by Sánchez-López et al. [45] showed that even in washed plant samples, not all dust particles were removed from the leaf surface, but their amount only decreased, leading to an overestimation of the HM concentration in plant tissues. It can be concluded that the procedure for preparation of plant samples is not standardized and requires additional elaboration.

Apart from the fact that each plant species has a specific ability to retain dust on the surface, dust deposition is also influenced by environmental factors [45], including climate (precipitations, wind strength), season, proximity to dust sources, etc.

#### 2.3.5. Biomass and Evaluation of Phytoremediation Applicability

To evaluate the success of phytoremediation, it is very important to consider the biomass. Two approaches are commonly used [24]: (1) using plant hyperaccumulators, that have high BCFs but low aboveground biomass, and (2) using non-hyperaccumulators, that produce high biomass. In this study, non-hyperaccumulators with multiple harvests in a single growing season were used, which may have great potential for phytoextraction of HMs according to Ali et al. [24]. The urban grassland area in Varaždin is mowed every two to three weeks, as needed, with at least 10 cuts during the growing season.

The fresh and dry biomass of the studied plant species is presented in Table 4. The dry biomass of *Taraxacum officinale*, *Plantago lanceolata* and *Trifolium repens* was estimated to be 0.65 t/ha, 2.79 t/ha, 1.83 t/ha and 5.0%, 21.5%, 14.1% of the total mass during one growing season, respectively. During the same period, all three species studied accounted for about 40% of the dry weight of the total harvest.

The results show that of the four heavy metals (Pb, Cu, Zn, Cd) with which the soils in the Varaždin area are most enriched, the largest mass can be removed of Zn with *Plantago lanceolata* (1.72 kg/ha year), while the smallest mass can be removed of Cd (0.0004 kg/ha year) with *Trifolim repens* (Table 4). Considering the required remediation time, the remediation of Zn, Cd, and Cu to background levels (only the top 5 cm of soil) would take several decades (25–56 years) with the three species studied, while it would take more than two thousand years for Pb, making it almost impossible (Table 4).

According to Peuke and Rennenberg [69], for significant pollutant reduction over 10 to 20 years, it is necessary to use plant species with a BCF of 20 and a biomass production of 10 t/ha or with a BCF of 10 and a biomass production of 20 t/ha. Vangronsveld et al. [21] state that phytoextraction should not take longer than 10 years to be economically feasible. Vangronsveld et al. [21] gave an overview of the calculated masses of certain HMs per unit area that a particular plant species can remove from contaminated soils, as well as biomasses of certain plant species and the required remediation time. For the well-known hyperaccumulator species *Thlaspi caerulescens* J. Presl & C. Presl, the following data were obtained: total Cd uptake: 2 kg/ha year, in another experiment 9% of total Cd with two crops; biomass production: 1 t/ha year, 2.6 t/ha year, even 5 t/ha year; cleanup time: five to nine harvests/years. For plants that produce a large biomass, such as some crops (maize, rapeseed, sunflower, tobacco) and fast-growing tree species (poplars and willows), the following data were obtained: total Cd uptake: 0.05–0.34 kg/ha year; biomass production: 2.4–20 t/ha year; and cleanup time: 58–255 years. In both examples, a higher Cd uptake was obtained than in this study. However, the data obtained by the various authors vary, and the inconsistent methodology makes an accurate comparison questionable.

Regarding the applicability of phytoremediation, most scientists agree that it is a promising technology for the remediation of polluted sites, but so far the practical effects are not so obvious. Limitations include the long period of time required for remediation, the limited number of contaminants that can be removed by a single plant species, the limited depth that can be reached by roots, the risk of HM entering the food web, the lack of knowledge, and the impossibility of controlling the process [23,28].

To increase the efficiency of phytoextraction, various agrotechnical measures such as fertilization, irrigation and liming to increase biomass production can also be applied [21]. Many scientists are investigating various soil amendments that can increase leaching, phytoavailability and plant uptake of heavy metals [21,23,70].

The economic feasibility of phytoextraction can be increased by combining it with phytomining [71], where certain metals can be extracted from the removed biomass (ash that can be used as ore), and the use of biomass as an energy source [23,27].

### 2.4. Possible Sources of Pollution

Pollution with HMs in urban areas, including the area of the city of Varaždin, can be explained on three levels: (1) general sources of pollution, (2) specific sources of pollution, and (3) the forms in which pollution occurs and is transmitted.

General sources of pollution include traffic, industry, floods, and various local sources of pollution that are not specifically analyzed in this study. Traffic in the Varaždin area includes road and rail traffic. Areas near railway stations, especially railway intersections with high traffic frequency, are known as places with high soil pollution with HMs [53,54,72,73]. However, these areas are very limited. Conversely, road traffic causes a lower intensity of soil pollution by HMs, but the whole urban area is intertwined with dense road networks, crossroads [11,52], and numerous parking lots [74], and is more or less affected by this pollution source. In the outskirts of Varaždin, there are several industries, including metal industry, which can be potential sources of pollution [3], although this study provides only weak evidence of this type of pollution. However, studies in other urban areas show higher concentrations of HMs near industrial areas [52]. In addition, the Drava River occasionally floods the outskirts of Varaždin. It is known that the water of the Drava is heavily polluted with Zn, Pb, and Cd, mainly due to historical Zn and Pb mining, smelting, and flotation, as well as wastewater discharged upstream (in Italy, Austria, and Slovenia) [75,76].

The probable specific sources of certain HMs taken from literature sources [72,75,77,78,79] are listed in Table 5. Since the determination of CFs and I_geo_, calculated in relation to the mean values of HMs for Europe, showed that soils in the Varaždin area are most enriched in Pb > Cu > Zn > Cd > Ni > Fe > Mn, the possible sources of HMs are presented in that order.

As indicated by the collected data from the literature, each HM may originate from more than one specific emission source [80]. In this case, the main emissions are assumed to come from friction of moving vehicle parts and combustion of gasoline and diesel fuel, while only locally from flooding.

It is interesting to note that, of all the HMs studied, the greatest anthropogenic enrichment of soil is in Pb, especially considering that Croatia is a member of the EU, where leaded gasoline has been banned under Directive 98/70/EC [81]. The considerable pollution can be explained by the fact that Pb is one of the less mobile elements in the soil, partly due to past pollution and partly still emitted by traffic. However, recent studies have shown that the introduction of unleaded gasoline has led to a decrease in Pb content in dust [52,82].

Dust and aerosols are the main forms of pollutant emission and transfer in urban areas [52,83]. They are generated by road and rail traffic, industry, domestic oil burning, construction and demolition activities, waste incineration, lifting of eroded soil particles from the surface that may contain HMs, etc. [84,85]. The distribution of air pollution is affected by wind direction and strength. Some of the dust deposited on the surface of buildings, transport infrastructure, and vegetation can be easily removed by wind. Another important form of pollution transfer is water, whether flood, rain, or storm runoff water. Sooner or later, the dust enters the soil through dry and wet deposition.

At 15 of the 16 investigated sites in Varaždin, dust and aerosols can be considered as the main forms of pollutant emission and transmission, since no floods have been recorded in the city area since 1972 due to the constructed dams and other hydrotechnical structures [86]. Only site 1 (Drava Island), located on the outskirts of the city, is occasionally flooded. The last one was recorded about half a year before sampling (in November 2012) [87].

In the city of Varaždin, in addition to deposition from the air, the intensity and amount of precipitation have a secondary effect on the distribution of HMs in the soil. Monthly and daily precipitation amounts for the period from January to July 2013 are shown in Figure S1. The total monthly precipitation amounts for January, February, March, April, May, June, and July 2013 are 122 mm, 128 mm, 113 mm, 62 mm, 95.5 mm, 60.4 mm, and 33.9 mm, respectively. The daily precipitation amounts are mostly below 20 mm, and the maximum daily precipitation amount of 33 mm was recorded on March 31. The precipitation amount per hour for the same period (Figure S2) shows their intensity. Most of the precipitation was light rain (< 2.5 mm/h), moderate rain (2.5–7.5 mm/h) occurred occasionally, and heavy rain (7.6–50 mm/h) was recorded only twice (Figure S2). As shown in the presented data (Figures S1 and S2), the highest monthly precipitation was recorded in the first three months of the year, and the precipitation intensity increases in the warmer season. The highest precipitation intensity that could affect the amount of HMs in dust deposited on the plant surface and in the soil was recorded on 24 June 2013 with 8 mm/h, and the third and fourth sampling days were 26 June 2013 and 2 July 2013.

Developing a hydrogeological conceptual model of the Varaždin alluvial aquifer for the period 2008–2017, Karlović et al. [88] reported that of the average annual precipitation, 21% is distributed as surface runoff, 34% as groundwater recharge, and 45% as actual evapotranspiration. About 50% of the average annual surface runoff occurs during the cold period from November to February.

## 3. Materials and Methods

### 3.1. Study Area

**Figure 6:**
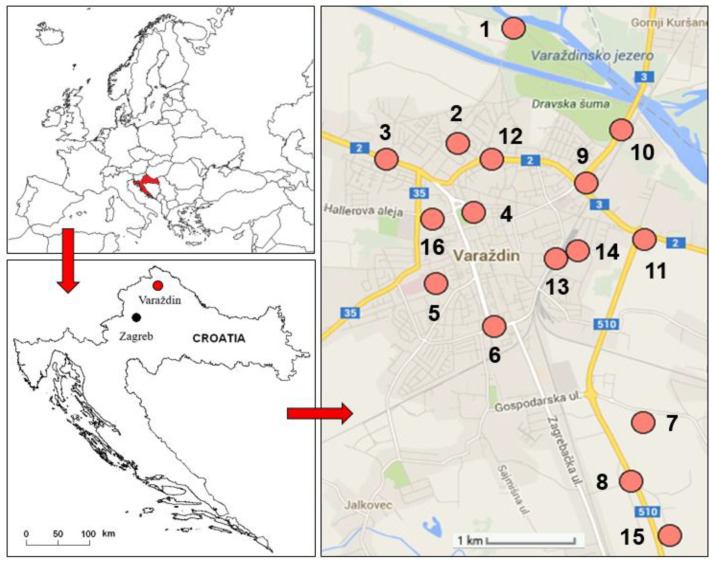
Study area of the city of Varaždin in northern Croatia with a position of 16 sites.

Field research was conducted in June and July 2013 at 16 sites in the city of Varaždin in northern Croatia (Figure 6). The city of Varaždin has about 39,000 inhabitants. It is located in the lowlands of the Drava River, at 16°20’33” east longitude and 46°18’29” north latitude, at an elevation of 169 to 175 m above sea level. The climate is temperate-continental, defined as C_fwbx_ type by Köppen classification and humid climate by Thornthwaite classification, with an average annual air temperature around 10 °C and with an average annual precipitation between 800 and 900 mm [89]. Data on precipitation per month, day and hour for the period from January to July 2013 were obtained from the Meteorological and Hydrological Service of Croatia in Zagreb [90].

### 3.2. Sampling and Sample Preparation

Soil and plant samples were collected from 16 sites (Figure 6). Site selection was based on locations with detected air pollution (unpublished data), different functional parts of the city, and different pollution sources and intensities: road traffic, parking lots, rail traffic, industrial areas, city parks, landfill, and floodplain. Composite soil samples were collected from urban grassland areas in the upper soil horizon (0–15 cm). Plant samples were collected from the same locations as the soil samples, cut 3–5 cm above the soil, and divided into two subsets.

In the first subset, three of the most abundant grassland plant species were sampled separately: *Taraxacum officinale*, *Plantago lanceolata*, and *Trifolium repens*. The plant samples were further divided into two subgroups. The first subgroup was laid out to dry, while the second subgroup was washed first with distilled water and then with deionized water (treated with Millipore DirectQ 3 Water Purification System, Molsheim, France) and laid out to dry. Dried washed and unwashed plant samples were later used for the determination of HMs. Additionally, the mass difference between the unwashed and washed plant samples was used to determine the heavy metal content in the dust deposited on the aboveground plant parts.

In the second subset, all plant species of 1 m^2^ were collected to determine plant biomass. The plant material was sorted into four groups immediately after field sampling: (i) *Taraxacum officinale*, (ii) *Plantago lanceolata*, (iii) *Trifolium repens*, and (iv) other plant species. Plant samples were weighed before and after drying. Then, the fresh biomass of the plants collected from an area of 1 m^2^ and the total concentration of heavy metals in the fresh plant and soil samples were used to calculate the mass of HMs that can be removed from the unit of area, and cleanup time.

The soil and plant samples were dried at room temperature to constant weight. Sample preparation included: grinding, sieving, and homogenization.

### 3.3. Determination of Soil pH

The soil reaction was determined in soil–water (pH_H2O_) and soil–salt (pH_KCl_) solution in suspension in the ratio 1: 2.5 (*w*/*v*) according to the following protocol: to 10 g of the soil sample, 25 mL of deionized water was added; and to 10 g of the soil sample, 25 mL of 1 M potassium chloride (KCl) solution was added; both suspensions mixed for 15 min with a magnetic stirrer and measured with a pH meter (SensION156, HACH Company, Loveland, CO, USA). Before measurement, the pH meter was calibrated with standard buffer solutions with pH values of 4.0, 7.0, and 10.0. Soil samples were classified by pH_H2O_ in ranges according to the classification accepted by FAO [91].

### 3.4. Determination of Soil Texture

Soil texture was determined according to the following protocol [92]: 25 mL of 0.1 M sodium pyrophosphate (Na_4_P_2_O_7_ 10H_2_O) was added to 10 g of air-dried specific soil fraction (< 2 mm particles), shaken, and left overnight. The next day, the fractions were separated by wet sieving through 212 µm and 63 µm sieves: coarse sand (2.0–0.2 mm) and fine sand (0.2–0.063 mm). A glass cylinder containing soil particles < 63 µm was filled up to 1000 mL with distilled water, the contents were shaken, and after 4 min and 48 s of sedimentation, 10 mL of soil suspension was pipetted from 10 cm depth and the fraction < 20 µm was obtained; after 4 h of sedimentation, 10 mL of suspension was pipetted again from 5 cm depth and the fraction < 2 µm was obtained. All separated fractions were dried in a thermostat at 105 °C to a constant mass, weighed, and the percentages of all fractions were calculated. Based on the percentages of the soil fractions, the soil samples were classified into texture classes [93].

### 3.5. Analysis of Organic Matter Content in Soil

The organic matter in the soil samples was determined by the volumetric method of Tjurin [94] as follows: 0.1–0.5 g of air-dried soil samples were weighed and mixed with 10 mL of 0.067 M potassium dichromate (K_2_Cr_2_O_7_) and 0.1 g of silver sulphate (Ag_2_SO_4_) and boiled in a digester for 5 min. The cooled sample was made up to a volume of 150 mL with distilled water, and 2 mL of a mixture of concentrated sulphate (H_2_SO_4_) and phosphoric acid (H_3_PO_4_) and diphenylamine sulfonic acid (C_12_H_11_NNaO_3_S) were added as an indicator. The amount of potassium dichromate used for the oxidation of the organic carbon was determined by titration with 0.1 M Mohr’s salt solution (Fe(NH_4_)_2_·6H_2_O), and the mass fraction of humus was calculated according to Equation (1):

(1) $$W_{\;\mathrm{humus}}\left(\%\right)=\frac{\left({\mathrm{Blank}\;\mathrm{titration}}_{\mathrm{Mohr}\;\mathrm{salt}\;\mathrm{depleted}}\;\left(\mathrm{mL}\right)-{\;\mathrm{Sample}\;\mathrm{titration}}_{\mathrm{Mohr}\;\mathrm{salt}\;\mathrm{depleted}}\;\left(\mathrm{mL}\right)\right)\times 0.0005172\;g\;}{\mathrm{soil}\;\mathrm{sample}\;\mathrm{mass}\;\left(g\right)}$$

The carbon content was calculated by dividing the humus content by a factor of 1.72.

### 3.6. Heavy Metal Analysis in Soils and Plants

Extraction of heavy metals from the soil samples was performed according to Marković [95] as follows: 15 mL of 36.5% hydrochloric acid (HCl) (*p.a.*, Kemika, Zagreb, Croatia) and 5 mL of 65% nitric acid (HNO_3_) (*p.a.*, Kemika, Zagreb, Croatia) were added to 3 g of each soil sample. Then, the samples were digested in a water bath at 50 °C for 6 h. After cooling, samples were filtered through filter paper (70 mm diameter, blue dot, Munktell-class 391) and diluted with deionized water to a volume of 50 mL. The solutions were further diluted with deionized water at a ratio of 1:5, 1:10, and 1:100.

Extraction of heavy metals from plant samples was performed according to the following protocol: 10 mL of 65% nitric acid (HNO_3_) (*p.a.*, Kemika, Zagreb, Croatia) was added to 1 g of each plant sample. Samples were digested in a water bath at 50 °C until the release of gaseous NO_2_ ceased. Then, 3 mL of 70% perchloric acid (HClO_4_) (*p.a.*, Kemika, Zagreb, Croatia) was added to the samples and digested again. The cooled samples were filtered through a filter paper (70 mm diameter, blue dot, Munktell-class 391) and diluted to a volume of 50 mL with deionized water. Depending on the metal content and calibration range, the solutions were further diluted with deionized water in ratios of 1:5, 1:10 and 1:100.

Heavy metals were detected by atomic absorption spectrometry (AAS) using an atomic absorption spectrometer (Perkin Elmer Analyst 800, 2006, PerkinElmer, Inc., Shelton, Connecticut, USA). Concentrations of Cd, Ni, and Pb were measured with a graphite (detection limits of 0.002 μg/L, 0.07 μg/L, and 0.05 μg/L, respectively), whereas concentrations of Cu, Fe, Mn, and Zn were measured with an air-acetylene flame (detection limits of 0.0015 μg/L for Cu, Mn, and Zn and of 5 μg/L for Fe). Hollow cathode lamps (HCL) were used as radiation sources for the detection of all heavy metals studied. For each heavy metal, the respective pure atomic spectroscopy-grade standard was used as reference material (PerkinElmer, Inc., Shelton, CO, USA).

### 3.7. Assesment of Soil Pollution and Plant Bioaccumulation

Contamination factors (CFs) were calculated as follows:

(2) $$\mathrm{CF}\;=\frac{C_{n\left(\mathrm{soil}\right)}}{B_{n\left(\mathrm{soil}\right)}}$$

Geoaccumulation indices (I_geo_) were calculated as follows:

(3) $$I_{\mathrm{geo}}={\;\log}_{2}\frac{C_{n\left(\mathrm{soil}\right)}}{1.5{\;B}_{n\left(\mathrm{soil}\right)}}$$

where C_n(soil)_ is the measured concentration of an element *n* in a soil sample, and B_n(soil)_ is the geochemical background concentration of the same element in soil reported by FOREGS [59]. There are six classes of I_geo_ [60].

Bioconcentration factors (BCFs) were calculated as follows:

(4) $$\mathrm{BCF}\;=\frac{C_{n\left(\mathrm{plant}\right)}}{{\;C}_{n\left(\mathrm{soil}\right)}}$$

where C_n(plant)_ is the measured concentration of an element *n* in a plant sample, and C_n(soil)_ is the measured concentration of the same element in the soil from the same location.

### 3.8. Statistical Analysis

Statistical analysis was performed using the STATISTICA 13.1 software package (StatSoft, Inc., Tulsa, OK, USA). Multivariate principal component analysis (PCA) was performed on the data matrices consisting of soil properties (humus content, carbon content, pH_H2O_, pH_KCl_, and grain size fractions) and total concentrations of HMs (Cd, Cu, Fe, Ni, Mn, Pb, and Zn) in both soils and plants. Correlation coefficients between different parameters were calculated, and the significance level was set at *p* < 0.05. The statistical significance of differences between heavy metal contents in unwashed and washed plant samples was determined by Mann–Whitney U test (*p* < 0.05).

## 4. Conclusions

According to the results of this study, the soil in the railway station in Varaždin (N Croatia) is the most polluted with heavy metals, but the area affected by railway traffic is relatively small. On the other hand, road traffic was found to pollute the soil to a lesser extent, but the entire city area is more or less affected by this source of pollution. All this requires further detailed studies and implementation of preventive measures to preserve the quality and ecological function of urban soils.

Dust, i.e., particulate matter generated by the friction of mowing vehicle parts and the combustion of fossil fuels, was determined as the main form in which heavy metals are emitted, transported, and deposited. Heavy precipitation and surface and soil water runoff are considered, at least in part, to be a factor affecting the spatial distribution of heavy metals in soils.

In investigating the possibility of removing heavy metals from polluted soils by phytoremediation, common wild species of urban grassland such as *Taraxacum officinale*, *Plantago lanceolata*, and *Trifolium repens* were used. These species have certain advantages: (i) they constitute a significant proportion of the total biomass of the urban grassland flora (about 40% dry weight); (ii) they provide a larger number of cuttings per year; (iii) they are naturally present in the urban grassland, i.e., there is no need to purchase seeds/seedlings or to sow/plant plants; and (iv) these species are also widespread in semi-natural grassland plant communities and in the composition of herbaceous vegetation in general. However, calculations based on the obtained data showed that phytoextraction of heavy metals from soil would take a long time with only the studied plant species. The problem lies not only in the plant species, but also in the mobility and bioavailability of the elements in question. In this study, a significant amount of organic matter and an alkaline soil reaction contributed to the immobilization of heavy metals that had accumulated in the upper soil horizon, making them less bioavailable. However, differences were also found between elements; e.g., lead, an element that was highly enriched in the soils, was also the one with the lowest bioaccumulation rate of all the elements studied.

Urban grasslands, while impacted by frequent mowing, trampling, and pollutant accumulation, provide numerous ecosystem services as part of the green infrastructure. One of these is their potential role in pollutant remediation, including phytostabilization and deposition, and due to increasing pressures from multiple sources, they need to be included in long-term monitoring programs.

## Figures and Tables

**Table 1:** Descriptive statistics of heavy metal content in soil samples and soil properties of studied sites of urban grassland in the city of Varaždin (N Croatia).

Heavy Metals and Soil Properties	Mean	Minimum	Maximum	Std. Dev.
Cd (mg/kg)	0.55	0.15	2.12	0.52
Cu (mg/kg)	46.1	24.8	161	32.7
Fe (g/kg)	28.9	20.5	52.0	8.204
Mn (mg/kg)	575	398	973	163
Ni (mg/kg)	37.2	19.2	73.1	12.3
Pb (mg/kg)	117	24.0	490	112
Zn (mg/kg)	148	55.8	481	112
Organic matter (%)	4.88	1.63	8.66	1.89
C (%)	2.84	0.95	5.03	1.10
pH_H20_	7.91	7.18	8.49	0.28
pH_KCl_	7.13	6.35	7.42	0.25
Coarse sand (%)	31.4	17.0	61.7	12.4
Fine sand (%)	21.0	13.0	32.0	5.67
Coarse silt (%)	20.1	6.70	25.2	4.59
Fine silt (%)	23.5	7.40	37.7	7.67
Clay (%)	3.96	1.00	7.10	1.47

**Table 2:** Descriptive statistics of heavy metal content, expressed in mg/kg, in washed plant samples (*Taraxacum officinale*, *Plantago lanceolata* and *Trifolium repens*) of urban grassland in the city of Varaždin (Northern Croatia).

Plant Species	Heavy Metals (mg/kg)	N	Mean	Minimum	Maximum	St. Dev.
*Taraxacum*	Cd	16	0.38	0.14	0.95	0.24
*officinale* agg.	Cu	16	15.1	8.5	23.5	4.0
(dandelion)	Fe	16	125	76	218	33
	Mn	16	34	22	50	9
	Ni	15	1.19	0.03	5.50	1.58
	Pb	16	0.24	0.06	0.91	0.29
	Zn	16	92	58	142	21
*Plantago*	Cd	15	0.25	0.13	0.63	0.14
*lanceolata* L.	Cu	14	9.8	7.1	18.8	2.9
(narrowleaf	Fe	15	139	86	246	52
plantain)	Mn	15	27	16	46	8
	Ni	15	0.95	0.30	1.86	0.51
	Pb	15	0.93	0.02	3.88	0.91
	Zn	14	104	66	186	39
*Trifolium*	Cd	15	0.03	0.01	0.04	0.01
*repens* L.	Cu	15	17.4	14.0	21.4	1.9
(white	Fe	15	138	85	201	33
clover)	Mn	15	25	16	32	5
	Ni	15	0.83	0.07	2.58	0.52
	Pb	15	0.52	0.13	1.52	0.45
	Zn	14	57	30	147	33

N—number of samples, St. Dev.—standard deviation

**Table 3:** Total mass of heavy metals (Cd, Cu, Fe, Mn, Ni, Pb and Zn) in dust deposited on the surface of *Taraxacum officinale*, *Plantago lanceolata* and *Trifolium repens*. The values given represent the difference between unwashed and washed plant samples.

	Cd	Cu	Fe	Mn	Ni	Pb	Zn
	mg/kg	mg/kg	mg/kg	mg/kg	mg/kg	mg/kg	mg/kg
*Taraxacum officinale* agg.							
Mean	−0.007 *	0.7	64	−4.1 *	−0.25 *	0.6	86
*Plantago lanceolata* L.							
Mean	−0.004 *	2.9	39	9.2	−0.07 *	1.0	26
*Trifolium repens* L.							
Mean	0.021	4.0	286	13.2	0.38	0.5	27
All three species							
N	46	45	46	46	45	44	44
Mean	0.003	2.5	128	5.9	0.02	0.7	48

*—The negative values are probably due to the fact that it was not possible to completely remove the dust particles from the plant samples by washing them with distilled and deionized water; N—number of samples.

**Table 4:** Average values of fresh and dry biomass of plant species collected from 16 sites from the Varaždin area, heavy metal removal and cleanup time.

	*Taraxacum officinale* agg.	*Plantago lanceolata* L.	*Trifolium repens* L.	Studied Species	All Other Species	Total Mass
**Fresh Biomass**						
single harvest-average (g/m^2^)	43.8	136	98.6	278	333	611
all harvests in growing season-average (g/m^2^)	410	1271	925	2606	3118	5723
all harvests in growing season-average (t/ha)	4.10	12.7	9.25	26.1	31.2	57.2
all harvests in growing season-average (%)	7.2	22.2	16.2	45.6	54.5	100
**Dry Biomass**						
single harvest-average (g/m^2^)	7.0	29.8	19.5	56.3	82.2	138
all harvests in growing season-average (g/m^2^)	65.2	279	183	527	771	1298
all harvests in growing season-average (t/ha)	0.65	2.79	1.83	5.27	7.71	13.0
all harvests in growing season-average (%)	5.00	21.5	14.1	40.6	59.4	100
**HMs Removal** (kg/ha year) *						
Cd	0.001	0.003	0.0004	0.0044		
Cu	0.073	0.167	0.208	0.448		
Pb	0.004	0.015	0.007	0.026		
Zn	0.663	1.72	0.447	2.83		
**Cleanup Time** (year) **						
Cd				37		
Cu				56		
Pb				>2000		
Zn				25		

*—HM removal was calculated as the ratio between the element concentration in the plant and its fresh biomass, expressed in kg/ha; **—Cleanup time was calculated as the ratio between the metal content in the first 5 cm of the soil, considering the soil density of 1.2 t/m^3^, and the metal content in the plant.

**Table 5:** Specific pollution sources in the area of Varaždin city.

	Pb	Cu	Zn	Cd	Ni	Fe	Mn
Road transport							
brake wear [77]							
tire wear [77]							
tire weights [78]							
road wear [77]							
Rail transport							
brakes [72]							
rails, wheels [72]							
galvanizing [72]							
Fuels and oils							
gasoline and diesel fuel [79]							
engine lubricating oil [79]							
Other sources							
floods [75]							

## Data Availability

Not applicable.

## Supplementary Materials

The following supporting information can be downloaded at: https://www.mdpi.com/article/10.3390/molecules27196558/s1, Table S1: All data collected at 16 sites in the area of the city of Varaždin (Northern Croatia) (name of the site, coordinates, date of collection of soil and plant samples, presumed source of pollution, number of mowing operations per year); results of laboratory analyses of soil and plant samples (soil fractions, humus content, soil reaction, heavy metals in soil and plant samples), mass of fresh and dry plant samples collected on one square meter and calculated bioaccumulation factors. Table S2: Descriptions and photographs of 16 sites in the area of the city of Varaždin (Northern Croatia) where soil and plant samples were collected. Table S3: Comparative table of HM values in the soil of various cities in Europe. Listed are countries, cities, literature sources, number of inhabitants, number of soil samples, depth from which the samples were collected, mean, minimum, and maximum concentrations of HMs (depending on availability). Table S4: Comparison of total concentrations of heavy metals in the area of Varaždin with maximum permissible values for the Republic of Croatia [55], target values [57] and intervention values [56] for the Netherlands. Numbers marked in red are values exceeding the maximum permissible values for Croatia [55]. Table S5: Contamination factors (CFs): (a) calculated in relation to the mean values of heavy metals in Croatia [58]; and (b) calculated in relation to the mean values of heavy metals for Europe [59]. Table S6: Geoaccumulation indices (I_geo_): (a) calculated in relation to the mean values of heavy metals in Croatia [58]; (b) calculated in relation to the mean values of heavy metals for Europe [59]; and (c) calculated in relation to the mean values of heavy metals for the region [59]. Table S7: Correlations between heavy metals in soil, soil pH, organic matter, carbon content, and soil fractions. Marked correlations are significant at *p* < 0.05, N = 16. Table S8: Comparative table with HM values in plant samples of *Taraxacum officinale*, *Plantago lanceoalta* and *Trifolium repens*. Listed are countries, locations, land use, time of sampling, types of plant samples, citations, number of samples, and mean, minimum and maximum values in mg/kg (where available). Table S9: Correlations between heavy metals in soil, plants, soil pH, organic matter, carbon content and soil fractions. Marked correlations are significant at *p* < 0.05, N = 16. Table S10: Comparative table of bioconcentration factors (BCFs) for the plant species *Taraxacum officinale*, *Plantago lanceolata*, and *Trifolium repens*, and for heavy metals (Cd, Cu, Fe, Mn, Ni, Pb, and Zn). Listed are countries, locations, land use, time of sampling, types of plant samples, citations, number of samples, and mean, minimum, and maximum values for PCBs (depending on availability). Table S11: Mann–Whitney U test with examined differences in heavy metal content between washed and unwashed plant samples of *Taraxacum officinale*, *Plantago lanceolata*, and *Trifolium repens*. Values marked in red are significant at *p* < 0.05. Figure S1. Total monthly precipitation and precipitation per day (mm) for the period from January to July 2013. Source of data: Meteorological and Hydrological Service of Croatia, Zagreb [90]. Figure S2. Precipitation amount per hour (mm) and intensity range for the period from January to July 2013. Source of data: Meteorological and Hydrological Service of Croatia, Zagreb [90].

## Sample Availability

Not available.
