# Supplementary material for: Can Urban Grassland Plants Contribute to the Phytoremediation of Soils Contaminated with Heavy Metals
Source: Molecules. 2022 Oct 4;27(19):6558. doi: 10.3390/molecules27196558 (PMC9572121; doi:10.3390/molecules27196558)
Supplement: Supplementary file 1 [file molecules-27-06558-s001.zip › molecules-1907113_Table S2-S11 and Figures S1 S2_ corrected version.pdf]

Table S2. Descriptions and photographs of 16 sites in the area of the city of Varaždin (Northern Croatia) where soil and plant samples were collected.

Site 1

Drava island, about 8 m from the forest and about 5 m from the macadam road, near the monument.

Observations: the stand is regularly mowed, part of the day in the shade, under the influence of floods.

Azimuth of the photograph: 175°.

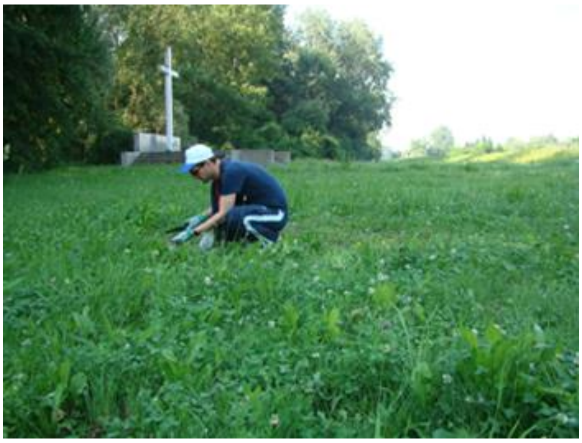

Site 2

Antun Gustav Matoš Square, about 18 m from the main road and about 8 m from the side road.

Observations: the stand is regularly mowed, the area is used as a park.

Azimuth of the photograph: 304°.

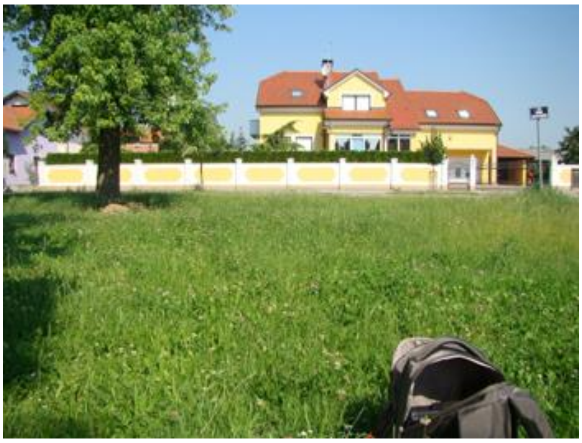

Site 3

Optujska Street, area between the church of St. Fabian and St. Sebastian and the parking lot of the former barracks, about 5 m from the entrance to the parking lot in front of the barracks.

Observations: the stand is mowed regularly, and is located on a busy road.

Azimuth of the photograph: 297°.

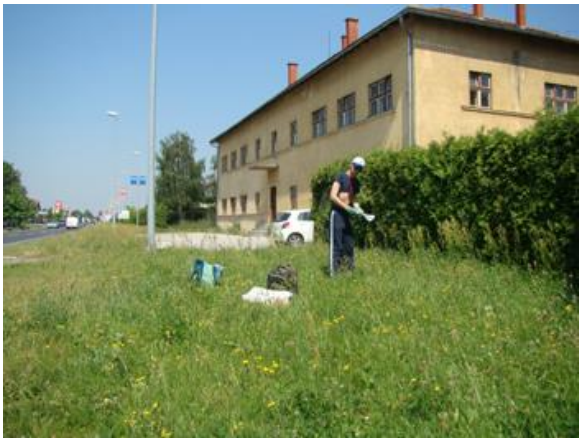

Site 4

Old town, between the kula and the dam.

Observations: location in flat terrain, lowest position in relation to the surrounding terrain, the stand is regularly mowed, the area is used as a park, protected from the influence of roads by a dam.

Azimuth of the photograph: 257°.

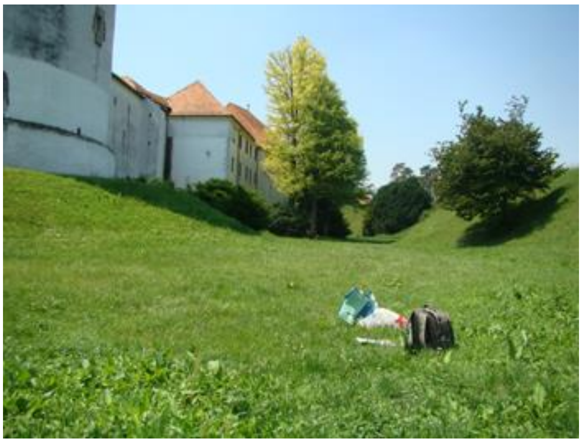

Site 5

General hospital, intersection of Meštrovićeva Street and F. Košćeca Street, about 8 m from Meštrovićeva Street and about 5 m from F. Košćeca Street.

Observations: the stand is regularly mowed, in the partial shade of trees (birch and maple), next to the parking lot.

Azimuth of the photograph: 322°.

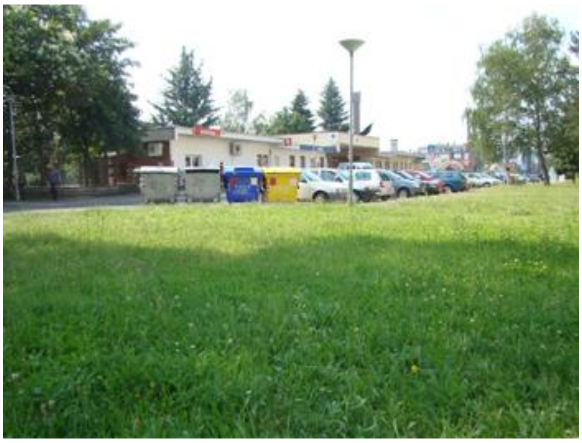

Site 6

Krleža Street, near the gas station.

Observations: the stand is regularly mowed, in the partial shade of trees, 3-4 m from a busy road.

Azimuth of the photograph: 72°.

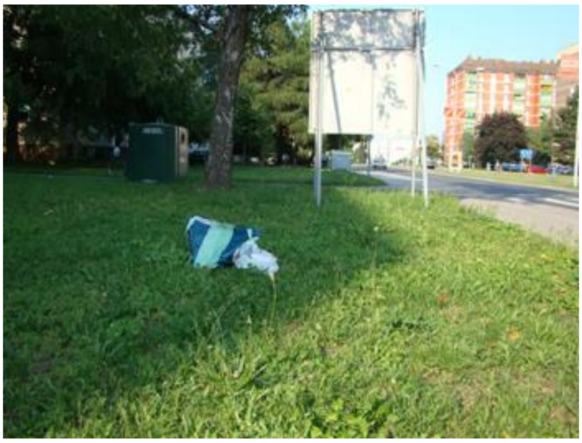

Site 7

Near Metal Industry Varaždin (MIV), Gospodarska Street, green area next to the HAK fence.

Observations: the stand in the industrial zone, along the road, is regularly mowed.

Azimuth of the photograph: 283°.

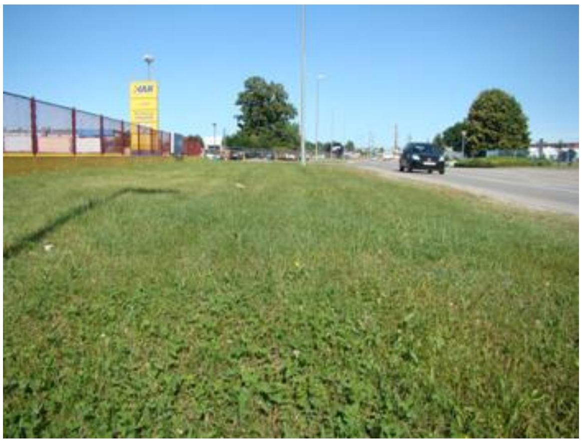

Site 8

Šilec gas station, Cehovska Street, green area between the gas station and the road.

Observations: the stand is mowed regularly, filled up about 10 years ago when the gas station was built.

Azimuth of the photograph: 333°.

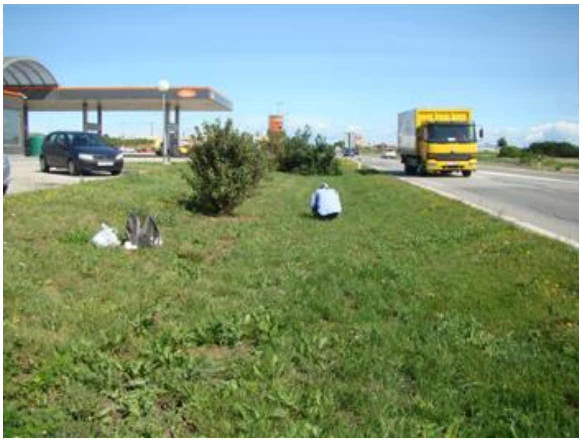

Site 9

Intersection of Koprivnička Street and Međimurska Street, a narrow green area between the road and the sidewalk.

Observations: the stand is regularly mowed, green area about 2 m wide, on a busy road.

Azimuth of the photograph: 334°.

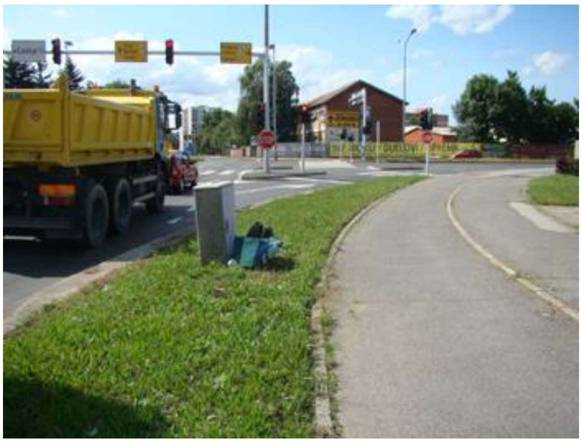

Site 10

Međimurska Street, near the bridge of Ban Josip Jelačić, green area between the main and side road.

Observations: the stand is regularly mowed, a green area between the roads is about 2 m wide.

Azimuth of the photograph: 20°.

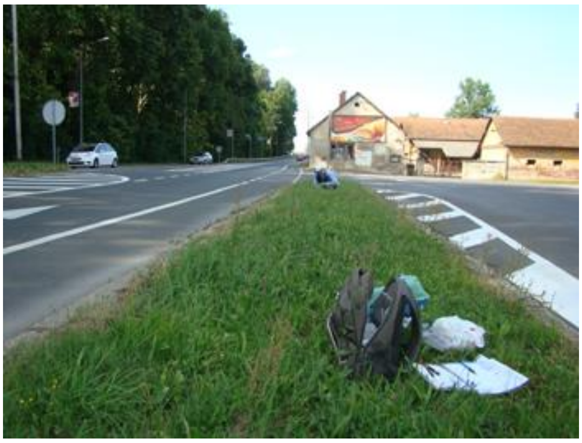

Site 11

Koprivnička Street, green area between the rotor and the road to Zagreb.

Observation: the stand is regularly mowed.

Azimuth of the photograph: 15°.

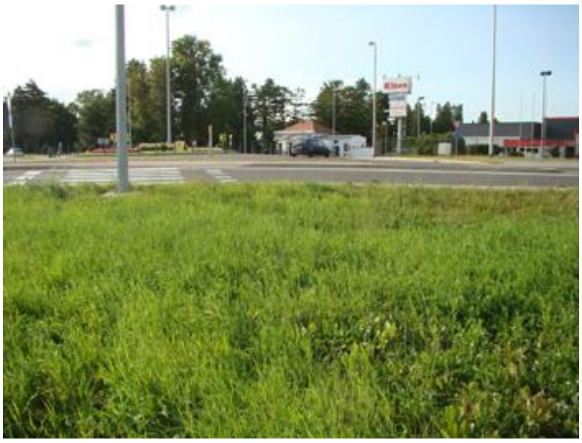

Site 12

Koprivnička Street, green area between roads, near the Petrol gas station on one side and the Kaufland shopping center on the other.

Observations: the stand is regularly mowed, the width of the green area is about 1.5 m.

Azimuth of the photograph: 84°.

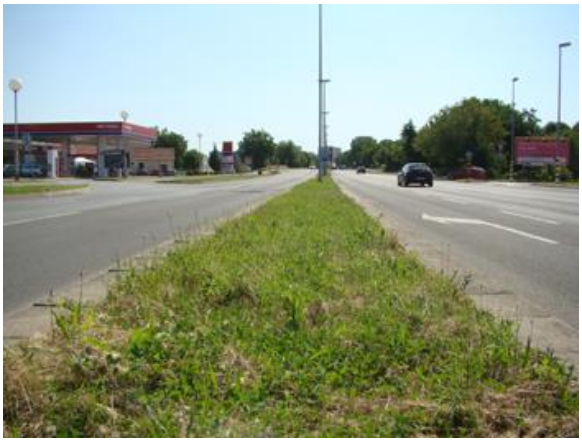

Site 13

Railway station Varaždin, near an abandoned building.

Observations: the width of the green area is about 2 m, the soil is mixed with stones, there is a lot of garbage on the surface.

Azimuth of the photograph: -

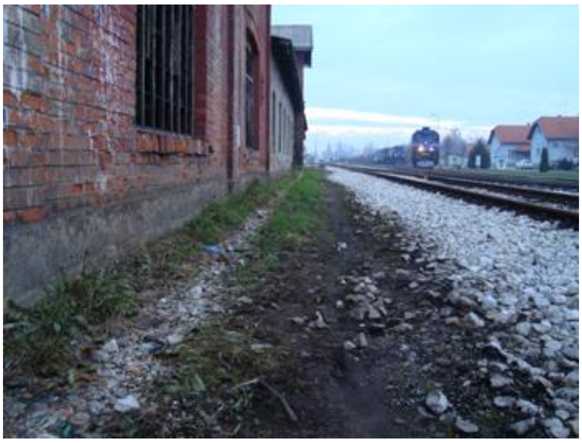

Site 14

Svilarska Street, along the fence of the factory VIS, near the entrance.

Observations: green area about 1.5 m wide, along a road with little traffic.

Azimuth of the photograph: 106°.

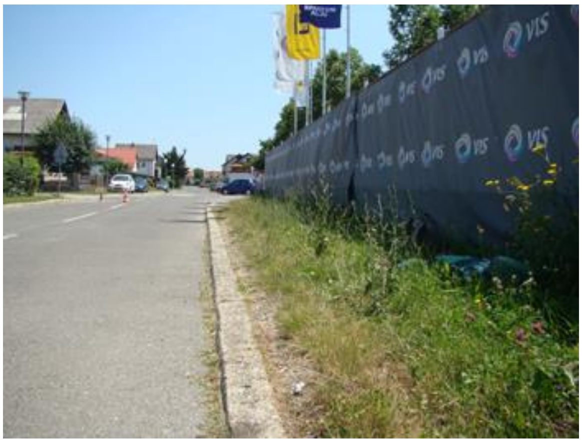

Site 15

Brezje, landfill, between the road and the waste bales.

Observations: green area about 2-3 m wide. Narrowleaf plantain and white clover were not found at this site.

Azimuth of the photograph: 191°.

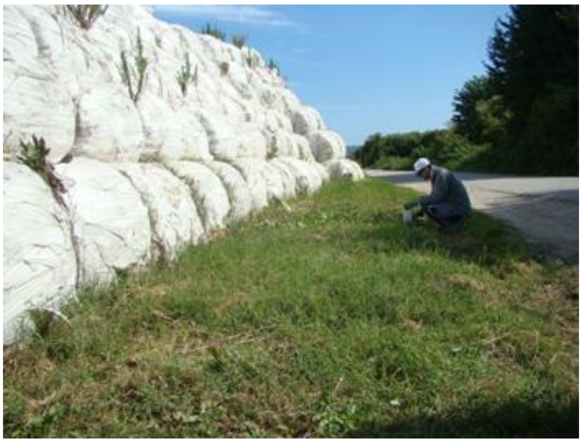

Site 16

Between the Faculty of Geotechnical Engineering and the Electromechanical School Varaždin, Haller's Alley.

Observations: the stand is regularly mowed, green area next to the parking lot.

Azimuth of the photograph: 0°.

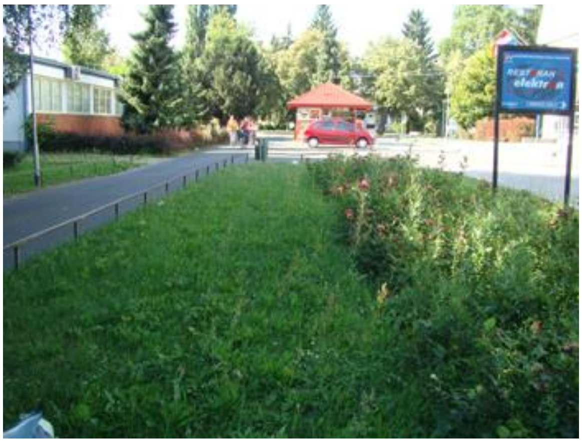

Table S3. Comparative table of HM values in the soil of various cities in Europe. Listed are countries, cities, literature sources, number of inhabitants, number of soil samples, depth from which the samples were collected, mean, minimum, and maximum concentrations of HMs (depending on availability).

Country and City	Number of Inhabitants	Number of Soil Samples	Soil Depth		Cd	Cu	Fe	Mn	Ni	Pb	Zn
								mg/kg			
Croatia, Varaždin – this study	39000	16	0-15 cm	Mean	0.6	46.1	28943	574.9	37.2	117.4	147.7
				Min.	0.2	24.8	20508	398.2	19.2	24.0	55.8
				Max.	2.1	160.8	51946	972.9	73.1	489.8	481.5
Slovenia, Celje – Leštan et al. [13]				Mean						294.2	1253
	37000	30	0-5 cm	Min.						43	170
				Max.						1690	6178
Slovenia, Celje – Finžgar et al. [12]	37000	6	0-5 cm	Mean						196.6	880.3
				Min.						56.3	163.3
				Max.						475.9	1564
Hungary, Sopron – Horváth et al. [5]	56000	104	0-10 cm	Mean	1.6	118.4			25.7	124.6	133.0
				Min.	0.4	11.5			5.5	27.6	26.8
				Max.	6.7	1221.0			98.7	558.7	606.5
Hungary, Szeged – Puskás and Farsang [6]	175300	15	0-10 cm	Mean		44.3			32.2	45.8	197.3
				Min.		25.7			16.6	22.7	136.8
				Max.		88.2			43.7	135.9	227.8
Serbia, Novi Sad – Škrbić and Čupić [9]	300000	6	0-5 cm	Mean	0.2	30.9	8009	251.3		10.8	85.5
				Min.	0.1	15.8	3112	66.6		31.6	41.4
				Max.	0.2	58.8	9668	311.7		0.2	255.5
Serbia, Belgrade – Crnković et al. [10]	1600000	46	0-10 cm	Mean		29.0			67.4	53.2	129.1
Austria, Wien (urban area, dynamic gradient) – Simon et al. [1]	> 2000000	n. a.	0-20 cm	Mean	0.2	18.0				65.0	97.0
Slovakia, Bratislava (crossroads) – Hiller et al. [11]	460000	39	0-10 cm	Mean		67.1	18242	419.0	20.9	54.2	174.0
Italy, Torino – Biasioli et al. [7]	1000000	70	0-20 cm	Mean		90.0			209.0	149.0	183.0

				Min.		34.0			103.0	31.0	78.0
				Max.		283.0			790.0	870.0	545.0
Germany, Pforzheim – Norra et al. [3]	115000	58	0-5 cm	Min.	< d. l.	6.6				15.8	13.9
				Max.	4.5	191.0				109.0	218.0
Germany, Berlin – Birke and Rauch [4]	> 3500000	2182	0-20 cm	Mean	0.9	79.5			10.7	119.0	243.0
				Max.	131.0	12300.0			769.0	4710.0	25210.0

Min. – Minimum, Max. – Maximum, n. a. – not available, d. l. – detection limit

Table S4. Comparison of total concentrations of heavy metals in the area of Varaždin with maximum permissible values for the Republic of Croatia [55], target values [57] and intervention values [56] for the Netherlands. Numbers marked in red are values exceeding the maximum permissible values for Croatia [55].

Site No.	Presumed Source of Pollution	Cd mg/kg	Cu mg/kg	Fe mg/kg	Mn mg/kg	Ni mg/kg	Pb mg/kg	Zn mg/kg
1	flood water	1.23 [NL]	49.6 [NL]	23307	492	30.0	121.1 [NL]	**357** [NL]
2	city park	0.37	38.5 [NL]	26506	548	38.7 [NL]	63.8	149 [NL]
3	road traffic	0.37	29.0	27147	973	53.6 [NL]	24.0	72
4	city park	0.45	74.1 [NL]	22841	487	31.8	133.8 [NL]	115
5	parking lot	0.41	39.5 [NL]	28064	811	33.7	53.8	112
6	road traffic	0.36	38.0 [NL]	26673	708	38.0 [NL]	63.0	133
7	industrial zone and road traffic	0.18	31.3	25182	438	26.3	45.0	87
8	gas station and road traffic	0.25	24.8	25140	398	19.2	50.5	56
9	road traffic	0.42	40.7 [NL]	22991	520	40.2 [NL]	96.5 [NL]	122
10	road traffic	0.48	32.7	27814	449	34.2	126.6 [NL]	101
11	road traffic	0.24	31.8	36577	661	35.3 [NL]	48.3	82
12	gas station and road traffic	0.33	45.8 [NL]	29730	467	36.2 [NL]	**204** [NL]	139
13	rail transport	**2.12** [NL]	**161** [NL]	51946	634	73.1 [NL]	**490** [NL]	**481** [NL]
14	industrial zone and road traffic	1.13 [NL]	37.7 [NL]	20508	400	43.3 [NL]	**156** [NL]	155 [NL]
15	landfill	0.15	26.5	25873	483	26.8	45.6	92
16	parking lot	0.24	37.0 [NL]	42783	728	34.2 [NL]	**157** [NL]	107
	Croatia – maximum permissible levels for arable land [55]	2	120	-	-	75	150	200
	The Netherlands – target values for soil/sediment [57]	0.8	36	-	-	35	85	140
	The Netherlands – intervention values for soil/sediment [56]	13	190	-	-	100	530	720

NL – over target value for the Netherlands.

Table S5. Contamination factors (CFs): a) calculated in relation to the mean values of heavy metals in Croatia [58]; and b) calculated in relation to the mean values of heavy metals for Europe [59].

	Site	CFs							
	**No.**	**Cd**	**Cu**	**Fe**	**Mn**	**Ni**	**Pb**	**Zn**	**Mean**
a)	1	1.8	1.7	0.7	0.6	0.5	3.2	3.6	1.7
	2	0.5	1.3	0.8	0.7	0.7	1.7	1.5	1.0
	3	0.5	1.0	0.8	1.2	1.0	0.6	0.7	0.8
	4	0.6	2.5	0.7	0.6	0.6	3.5	1.2	1.4
	5	0.6	1.3	0.8	1.0	0.6	1.4	1.1	1.0
	6	0.5	1.3	0.8	0.9	0.7	1.7	1.3	1.0
	7	0.3	1.0	0.7	0.5	0.5	1.2	0.9	0.7
	8	0.4	0.8	0.7	0.5	0.3	1.3	0.6	0.7
	9	0.6	1.4	0.7	0.6	0.7	2.5	1.2	1.1
	10	0.7	1.1	0.8	0.6	0.6	3.3	1.0	1.2
	11	0.3	1.1	1.0	0.8	0.6	1.3	0.8	0.9
	12	0.5	1.5	0.8	0.6	0.7	5.4	1.4	1.6
	13	3.0	5.4	1.5	0.8	1.3	12.9	4.9	4.2
	14	1.6	1.3	0.6	0.5	0.8	4.1	1.6	1.5
	15	0.2	0.9	0.7	0.6	0.5	1.2	0.9	0.7
	16	0.3	1.2	1.2	0.9	0.6	4.1	1.1	1.4
	Mean	0.8	1.5	0.8	0.7	0.7	3.1	1.5	
b)	1	4.4	2.9	0.6	0.6	0.8	3.7	5.2	2.6
	2	1.3	2.2	0.7	0.7	1.0	2.0	2.2	1.4
	3	1.3	1.7	0.7	1.2	1.4	0.7	1.1	1.2
	4	1.6	4.3	0.6	0.6	0.9	4.1	1.7	2.0
	5	1.5	2.3	0.7	1.0	0.9	1.7	1.7	1.4
	6	1.3	2.2	0.7	0.9	1.0	1.9	2.0	1.4
	7	0.6	1.8	0.7	0.5	0.7	1.4	1.3	1.0
	8	0.9	1.4	0.7	0.5	0.5	1.5	0.8	0.9
	9	1.5	2.3	0.6	0.6	1.1	3.0	1.8	1.6
	10	1.7	1.9	0.7	0.6	0.9	3.9	1.5	1.6
	11	0.9	1.8	1.0	0.8	0.9	1.5	1.2	1.2
	12	1.2	2.6	0.8	0.6	1.0	6.3	2.0	2.1
	13	7.6	9.3	1.4	0.8	2.0	15.0	7.1	6.2
	14	4.0	2.2	0.5	0.5	1.2	4.8	2.3	2.2
	15	0.5	1.5	0.7	0.6	0.7	1.4	1.4	1.0
	16	0.9	2.1	1.1	0.9	0.9	4.8	1.6	1.8
	Mean	1.9	2.7	0.8	0.7	1.0	3.6	2.2	
	CFs:	1-.4.9	5-9.9	>10					

Table S6. Geoaccumulation indices (I_{geo}): a) calculated in relation to the mean values of heavy metals in Croatia [58]; b) calculated in relation to the mean values of heavy metals for Europe [59]; and c) calculated in relation to the mean values of heavy metals for the region [59].

		I_{geo}						
	**Site No.**	**Cd**	**Cu**	**Fe**	**Mn**	**Ni**	**Pb**	**Zn**
a)	1	0.2	0.1	-1.2	-1.3	-1.5	1.1	1.3
	2	-1.5	-0.2	-1.0	-1.1	-1.1	0.2	0.0
	3	-1.5	-0.6	-1.0	-0.3	-0.6	-1.2	-1.0
	4	-1.2	0.7	-1.2	-1.3	-1.4	1.2	-0.4
	5	-1.4	-0.2	-0.9	-0.6	-1.3	-0.1	-0.4
	6	-1.6	-0.2	-1.0	-0.8	-1.1	0.1	-0.2
	7	-2.5	-0.5	-1.1	-1.5	-1.6	-0.3	-0.8
	8	-2.1	-0.9	-1.1	-1.6	-2.1	-0.2	-1.4
	9	-1.3	-0.1	-1.2	-1.2	-1.0	0.8	-0.3
	10	-1.1	-0.5	-0.9	-1.4	-1.3	1.2	-0.6
	11	-2.1	-0.5	-0.5	-0.9	-1.2	-0.2	-0.8
	12	-1.6	0.0	-0.8	-1.4	-1.2	1.8	-0.1
	13	1.0	1.8	0.0	-0.9	-0.2	3.1	1.7
	14	0.1	-0.3	-1.4	-1.6	-0.9	1.4	0.1
	15	-2.8	-0.8	-1.0	-1.3	-1.6	-0.3	-0.7
	16	-2.1	-0.3	-0.3	-0.7	-1.3	1.5	-0.5
	Mean	-1.3	-0.1	-0.9	-1.1	-1.2	0.6	-0.3
b)	1	1.6	0.9	-1.3	-1.3	-0.9	1.3	1.8
	2	-0.2	0.6	-1.1	-1.1	-0.5	0.4	0.5
	3	-0.2	0.2	-1.1	-0.3	-0.1	-1.0	-0.5
	4	0.1	1.5	-1.3	-1.3	-0.8	1.5	0.2
	5	0.0	0.6	-1.0	-0.6	-0.7	0.1	0.1
	6	-0.2	0.5	-1.1	-0.8	-0.6	0.4	0.4
	7	-1.2	0.3	-1.2	-1.5	-1.1	-0.1	-0.2
	8	-0.8	-0.1	-1.2	-1.6	-1.5	0.0	-0.9
	9	0.0	0.6	-1.3	-1.2	-0.5	1.0	0.3
	10	0.2	0.3	-1.0	-1.4	-0.7	1.4	0.0
	11	-0.8	0.3	-0.6	-0.9	-0.7	0.0	-0.3
	12	-0.3	0.8	-0.9	-1.4	-0.6	2.1	0.4
	13	2.3	2.6	-0.1	-0.9	0.4	3.3	2.2
	14	1.4	0.5	-1.5	-1.6	-0.4	1.7	0.6
	15	-1.5	0.0	-1.1	-1.3	-1.1	-0.1	-0.1
	16	-0.8	0.5	-0.4	-0.7	-0.7	1.7	0.1
	Mean	0.0	0.6	-1.0	-1.1	-0.7	0.8	0.3
c)	1	2.6	0.6	-1.5	-1.2	-0.7	1.5	1.5
	2	0.8	0.2	-1.3	-1.1	-0.3	0.6	0.3
	3	0.8	-0.2	-1.3	-0.3	0.2	-0.9	-0.8
	4	1.1	1.2	-1.5	-1.3	-0.6	1.6	-0.1
	5	1.0	0.3	-1.3	-0.5	-0.5	0.3	-0.1
	6	0.8	0.2	-1.3	-0.7	-0.3	0.5	0.1
	7	-0.2	-0.1	-1.4	-1.4	-0.9	0.0	-0.5

8	0.2	-0.4	-1.4	-1.5	-1.3	0.2	-1.2
9	1.0	0.3	-1.5	-1.2	-0.3	1.1	0.0
10	1.2	0.0	-1.3	-1.4	-0.5	1.5	-0.3
11	0.2	-0.1	-0.9	-0.8	-0.4	0.2	-0.6
12	0.7	0.5	-1.2	-1.3	-0.4	2.2	0.2
13	3.3	2.3	-0.4	-0.9	0.6	3.5	2.0
14	2.4	0.2	-1.7	-1.5	-0.1	1.8	0.3
15	-0.5	-0.3	-1.4	-1.3	-0.8	0.1	-0.4
16	0.2	0.2	-0.6	-0.7	-0.5	1.8	-0.2
Mean	1.0	0.3	-1.2	-1.1	-0.4	1.0	0.0

Six classes of I_{geo} [60]:
practically uncontaminated (I_{geo} <= 0),
uncontaminated to moderately contaminated (0 < Igeo < 1),
moderately contaminated (1 < I_{geo} < 2),
moderately to heavily contaminated (2 < I_{geo} < 3),
heavily contaminated (3 < I_{geo} < 4),
heavily to extremely contaminated (4 < I_{geo} < 5),
extremely contaminated (I_{geo} > 5).

Table S7. Correlations between heavy metals in soil, soil pH, organic matter, carbon content, and soil fractions. Marked correlations are significant at $p < 0.05$, N=16.

	C	pH_{H20}	pH_{KCl}	Cd	Cu	Fe	Ni	Mn	Pb	Zn	Clay	Coarse sand	Fine sand	Coarse silt	Fine silt
**Humus**	**1**	-0.32	-0.03	**0.61**	0.46	0.21	0.15	-0.38	**0.54**	0.49	0.25	0.13	-0.37	0.00	0.02
**C**		-0.32	-0.03	**0.61**	0.46	0.21	0.15	-0.38	**0.54**	0.49	0.25	0.13	-0.37	-0.01	0.02
**pH_{H20}**			**0.72**	-0.01	-0.12	0.02	0.23	-0.07	0.03	-0.09	-0.17	0.00	0.49	-0.04	-0.31
**pH_{KCl}**				0.19	0.14	-0.01	0.16	-0.43	0.26	0.22	-0.22	-0.02	0.45	0.00	-0.26
**Cd**					**0.83**	0.42	**0.71**	-0.06	**0.83**	**0.93**	-0.07	0.26	-0.09	-0.12	-0.27
**Cu**						**0.65**	**0.74**	0.04	**0.91**	**0.83**	-0.18	0.23	-0.23	-0.07	-0.12
**Fe**							**0.58**	0.36	**0.69**	0.49	-0.09	0.25	-0.33	-0.18	-0.03
**Ni**								0.44	**0.71**	**0.62**	-0.36	0.29	-0.01	-0.18	-0.28
**Mn**									-0.09	-0.04	-0.07	0.23	-0.36	-0.23	0.04
**Pb**										**0.81**	-0.12	0.09	-0.07	0.05	-0.09
**Zn**											-0.06	0.19	-0.11	-0.06	-0.17
**Clay**												**-0.60**	-0.50	**0.63**	**0.78**
**Coarse sand**													-0.15	**-0.95**	**-0.82**
**Fine sand**														0.02	-0.41
**Coarse silt**															**0.80**

Table S8. Comparative table with HM values in plant samples of *Taraxacum officinale*, *Plantago lanceoalta* and *Trifolium repens*. Listed are countries, locations, land use, time of sampling, types of plant samples, citations, number of samples, and mean, minimum and maximum values in mg/kg (where available).

**Country, Location/s, Land Use, Sampling Time, Plant Samples, Citation**	**Cd**<br>N<br>mean mg/kg<br>(range mg/kg)	**Cu**<br>N<br>mean mg/kg<br>(range mg/kg)	**Fe**<br>N<br>mean mg/kg<br>(range mg/kg)	**Mn**<br>N<br>mean mg/kg<br>(range mg/kg)	**Ni**<br>N<br>mean mg/kg<br>(range mg/kg)	**Pb**<br>N<br>mean mg/kg<br>(range mg/kg)	**Zn**<br>N<br>mean mg/kg<br>(range mg/kg)
Taraxacum officinale							
Bosnia and Herzegovina (Cazin, Fojnica, Goražde, Počitelj, Sarajevo, Živinice), along the roads, May 2008, leaves DW, wild plants, Šaćiragić-Borić [30]	12<br>0.53 (0.29-0.75)	-<br>-	-<br>-	-<br>-	-<br>-	12<br>5.63 (0.45-14.22)	12<br>44.2 (23.5-61.6)
Bulgaria (Sofia), residential area and city park, April - November, leaves DW, wild plants, Djingova and Kuleff [31]	14<br>0.45 (<0.1-1.30)	14<br>12 (4-22)	-<br>-	14<br>21 (4.4-33)	-<br>-	14<br>1.5 (<0.1-5.2)	14<br>56 (17-90)
Italy (Imperina Valley), abandoned mined area (Cu and S), spring 2011, leaves DW, wild plants, Bini et al. [32]	4<br>0.86 (0.29-1.46)	4<br>41.8 (9-64)	4<br>617.5 (470-890)	-<br>-	-<br>-	4<br>115.8 (3.3-193)	4<br>112 (44-189)
Italy (Rome), city area, along the roads, March, June and October 1999, leaves DW, wild plants, Malizia et al. [33]	-<br>-	15<br>33 (10-52)	-<br>-	15<br>50 (19-98)	-<br>-	11<br>11 (1-28)	15<br>97 (40-150)
Italy (Piedmont region: Alba, Bra, Cherasco, Cuneo, Entracque, Fossano, Genola, Marene, Saluzzo, Sanfrè, Sant'Albano Stura), traffic impacted rural areas, time of sampling not specified, leaves DW, wild plants, Giacomino et al. [34]	13<br>0.19 (0.09-0.39)	13<br>12.4 (5.1-28.8)	13<br>361 (69.5-2632)	13<br>48.0 (23.3-170)	-<br>-	13<br>0.61 (0.23-2.27)	13<br>42.6 (21.4-70.6)
Poland (the whole country), located as far away as possible from point sources of pollution, middle of July 1982-1986, leaves DW, wild plants, Kabata-Pendias and Dudka [35]	150<br>0.69 (0.2-5.0)	150<br>11.8 (4-21.6)	150<br>184.4 (44-900)	150<br>51.45 (4-500)	150<br>1.9 (0.4-8.8)	150<br>3.53 (0.8-10.0)	150<br>45.1 (20.2-230.0)
Poland (the whole country), May 1993 and 1994, leaves DW, wild plants, Kabata-Pendias and Krakowiak [36]	780<br>0.5	780<br>9.4	780<br>241	780<br>60	780<br>3.4	780<br>1.1	780<br>45
Poland (Poznan), city - along the roads, May and July 1998, leaves DW, wild plants, Diatta et al. [37]	29<br>1.3 (0.6-2.2)	29<br>15.5 (5.7-58.2)	-<br>-	-<br>-	29<br>4.5 (1.5-8.7)	29<br>14.7 (1.7-36.1)	29<br>65.5 (18.5-140.4)
Poland (Cracow – Zakopane), along the road, September 2006, leaves DW, wild plants, Korzeniowska and Panek [38]	35<br>1.7 (0.1-6.1)	35<br>14.4 (8.5-37.2)	-<br>-	-<br>-	35<br>4.6 (0.3-14.4)	35<br>3.3 (0.3-39.8)	35<br>49.5 (24.0-106.0)
**Summarised**	**1037**<br>**0.59 (0.09-6.1)**	**1040**<br>**10.6 (4-64)**	**947**<br>**235 (44-2632)**	**972**<br>**57.8 (4-500)**	**994**<br>**3.3 (0.3-14.4)**	**1048**<br>**2.5 (<0.1-193)**	**1052**<br>**46.8 (17-230)**
**Varaždin, Croatia, this study**	**16**	**16**	**16**	**16**	**15**	**16**	**16**

	**0.38 (0.14-0.95)**	**15.1 (8.50-23.5)**	**125 (75.7-218)**	**33.7 (22.4-49.6)**	**1.19 (0.03-5.50)**	**0.24 (0.06-0.91)**	**92.1 (58.3-142)**
Plantago lanceolata							
Bulgaria (Sofia, Losenetz), residential area and city park, April –	13	13	-	13	-	13	13
November, leaves DW, wild plants, Djingova and Kuleff [31]	0.16 (<0.08-0.33)	8.2 (2-16.4)	-	18 (7.7-29.2)	-	2.6 (1.2-5.9)	32 (16-49)
Slovenia (Celje), city and zinc smelter, time of sampling not	-	-	-	-	-	25	25
specified, leaves, DW, wild plants, Leštan et al. [13]	-	-	-	-	-	3.0 (0.7-13.1)	156 (43-609)
Poland (Lublin region: Branica Radzyńska), natural clean area,	10	10	10	10	-	10	10
edge of a forest, June 2003 and 2004, leaves DW, wild plants, Sembratowicz et al. [39]	0.23 (0.1-0.33)	9.5 (6-15)	11.5 (7-17)	22 (12-30)	-	1.32 (0.54-2.23)	40 (32-50)
**Summarised**	**23**	**23**	**10**	**23**	-	**48**	**48**
	**0.19 (<0.08-0.33)**	**8.8 (2-16.4)**	**11.5 (7-17)**	**19.7 (7.7-30)**	-	**2.57 (0.54-13.1)**	**98 (16-609)**
**Varaždin, Croatia, this study**	**15**	**14**	**15**	**15**	**15**	**15**	**14**
	**0.25 (0.13-0.63)**	**9.83 (7.13-18.8)**	**139 (86.3-246)**	**27.4 (16.2-45.6)**	**0.95 (0.30-1.86)**	**0.93 (0.02-3.88)**	**104 (66.05-186)**
Trifoilum repens							
Bosnia and Herzegovina (Spreča river valley, Donja Lohinja),	3	3	-	-	3	3	3
flooding area, March 2020, above-ground plant parts DW, native plants, Murtić et al. [40]	0.03	16.5	-	-	88.2	2.43	31.3
France, closed lead smelter, October 2004, above-ground parts,	9	-	-	-	-	9	9
DW, sown plants, Bidar et al. [41]	9.01	-	-	-	-	35.8	96.7
France, closed lead smelter, autumn 2004, spring and autumn	3	-	-	-	-	3	3
2005, above-ground parts, DW, sown plants, Bidar et al. [42]	5.0 (3.5-7.8)	-	-	-	-	35.7 (18-59.3)	138.9 (87.8-168)
**Summarised**	**15**	**3**	-	-	**3**	**15**	**15**
	**6.41 (0.033-9.01)**	**16.46**	-	-	**88.2**	**29.1 (2.43-59.3)**	**92 (31-168)**
**Varaždin, Croatia, this study**	**15**	**15**	**15**	**15**	**15**	**15**	**14**
	**0.03 (0.01-0.04)**	**17.4 (14.0-21.4)**	**138 (84.7-201)**	**24.7 (15.7-32.0)**	**0.83 (0.07-2.58)**	**0.52 (0.13-1.52)**	**56.7 (30.2-147)**

N – number of samples, DW – dry weight

Table S9. Correlations between heavy metals in soil, plants, soil pH, organic matter, carbon content and soil fractions. Marked correlations are significant at $p < 0.05$, N=16.

	**Silt (%)**	**Humus**	**C**	**pH_{H20}**	**pH_{KCl}**	**Cd_{soil}**	**Cu_{soil}**	**Fe_{soil}**	**Ni_{soil}**	**Mn_{soil}**	**Pb_{soil}**	**Zn_{soil}**	**Cd_{plants}**	**Cu_{plants}**	**Fe_{plants}**	**Ni_{plants}**	**Mn_{plants}**	**Pb_{plants}**	**Zn_{plants}**
**Sand (%)**	-1.00	-0.04	-0.04	0.22	0.14	0.31	0.21	0.09	0.36	0.12	0.13	0.21	-0.08	-0.02	-0.04	-0.12	-0.19	-0.04	-0.14
**Silt (%)**		0.01	0.01	-0.22	-0.13	-0.32	-0.19	-0.08	-0.34	-0.12	-0.12	-0.22	0.10	0.04	0.06	0.13	0.20	0.03	0.15
**Humus**			1.00	-0.38	-0.03	0.58	0.48	0.27	0.11	-0.38	0.55	0.50	0.05	0.09	0.33	-0.11	-0.15	0.13	0.17
**C**				-0.38	-0.03	0.58	0.48	0.27	0.11	-0.38	0.55	0.51	0.05	0.09	0.33	-0.12	-0.15	0.13	0.17
**pH_{H20}**					0.68	-0.02	-0.14	0.02	0.24	0.03	-0.01	-0.11	0.07	0.10	0.09	0.04	0.05	0.09	-0.06
**pH_{KCl}**						0.25	0.19	0.03	0.21	-0.35	0.29	0.26	0.18	0.31	0.23	-0.02	-0.05	0.15	0.04
**Cd_{soil}**							0.84	0.48	0.70	-0.05	0.84	0.94	0.21	0.09	0.29	-0.16	-0.26	0.36	0.06
**Cu_{soil}**								0.66	0.74	0.03	0.92	0.83	0.27	0.23	0.39	-0.15	-0.27	0.34	-0.04
**Fe_{soil}**									0.61	0.35	0.74	0.51	0.20	0.22	0.34	-0.17	-0.02	0.37	-0.17
**Ni_{soil}**										0.47	0.71	0.61	0.25	0.12	0.33	-0.20	-0.17	0.28	-0.01
**Mn_{soil}**											-0.07	-0.04	-0.07	-0.22	-0.20	-0.29	-0.09	0.01	-0.25
**Pb_{soil}**												0.81	0.31	0.31	0.46	-0.13	-0.11	0.37	0.04
**Zn_{soil}**													0.24	0.12	0.25	-0.14	-0.25	0.36	0.01
**Cd_{plants}**														0.10	0.21	0.14	0.38	-0.20	0.46
**Cu_{plants}**															0.44	0.17	0.29	-0.12	-0.18
**Fe_{plants}**																0.29	0.29	0.16	0.14
**Ni_{plants}**																	0.48	0.20	0.28
**Mn_{plants}**																		-0.16	0.29
**Pb_{plants}**																			0.05

Table S10. Comparative table of bioconcentration factors (BCFs) for the plant species *Taraxacum officinale*, *Plantago lanceolata*, and *Trifolium repens*, and for heavy metals (Cd, Cu, Fe, Mn, Ni, Pb, and Zn). Listed are countries, locations, land use, time of sampling, types of plant samples, citations, number of samples, and mean, minimum, and maximum values for PCBs (depending on availability).

Country, Location/s, Land Use, Sampling Time, Plant Samples, Citation	**Cd**<br>N<br>BCF – mean (range)	**Cu**<br>N<br>BCF – mean (range)	**Fe**<br>N<br>BCF – mean (range)	**Mn**<br>N<br>BCF – mean (range)	**Ni**<br>N<br>BCF – mean	**Pb**<br>N<br>BCF – mean (range)	**Zn**<br>N<br>BCF – mean (range)
Taraxacum officinale							
Italy, Imperina Valley, abandoned mined area (Cu and S), spring 2011, leaves DW, wild plants, Bini et al. [32]	4<br>1.63 (0.07-4.56)	4<br>0.04 (0.01-0.09)	4<br>0.007 (0.002-0.012)			4<br>0.03 (0.01-0.085)	4<br>0.17 (0.05-0.46)
Italy, Rome, city area, along the roads, March, June and October 1999, leaves DW, wild plants, Malizia et al. [33]	-	15<br>0.32 (0.18-0.47)		15<br>0.08 (0.03-0.12)		11<br>0.02 (0.005-0.036)	15<br>0.45 (0.09-1.148)
Italy, Piedmont region (Alba, Bra, Cherasco, Cuneo, Entracque, Fossano, Genola, Marene, Saluzzo, Sanfrè, Sant'Albano Stura), traffic impacted rural areas, time of sampling not specified, leaves DW, wild plants, Giacomino et al. [34]	13<br>0.84	13<br>0.45	13<br>0.016	13<br>0.08		13<br>0.025	13<br>0.47
Poland, Poznan, city - along the roads, May and July 1998, leaves DW, wild plants, Diatta et al. [37]	29<br>2.99	29<br>0.76			29<br>1	29<br>0.14	29<br>0.76
**Summarised**	**46**<br>**2.26 (0.07-4.56)**	**61**<br>**0.54 (0.01-0.76)**	**17**<br>**0.014**	**28**<br>**0.08**	**29**<br>**1**	**57**<br>**0.08**	**61**<br>**0.58**
**Varaždin, Croatia, this study**	**16**<br>**0.96 (0.22-2.11)**	**16**<br>**0.39 (0.15-0.69)**	**16**<br>**0.005 (0.003-0.009)**	**16**<br>**0.06 (0.03-0.12)**	**15**<br>**0.04 (0.001-0.21)**	**16**<br>**0.003 (0.0002-0.02)**	**16**<br>**0.83 (0.16-1.70)**
Plantago lanceolata							
Bulgaria, Plovdiv, city, July 1998 and 2001, leaves DW, wild Plants, Dimitrova and Yurukova [67]	8<br>0.34 (0.22-0.53)	8<br>0.15 (0.04-0.30)				8<br>0.19 (0.04-0.54)	8<br>0.47 (0.26-0.77)
Slovenia, Celje, city and zinc smelter, time of sampling not specified, leaves, DW, wild plants, Leštan et al. [13]	-					25<br>0.014 (0.002-0.075)	25<br>0.21 (0.02-0.77
**Summarised**	**8**<br>**0.34**	**8**<br>**0.15**	**-**	**-**	**-**	**33**<br>**0.057**	**33**<br>**0.27**
**Varaždin, Croatia, this study**	**15**<br>**0.63 (0.15-1.89)**	**14**<br>**0.25 (0.05-0.46)**	**15**<br>**0.005 (0.003-0.011)**	**15**<br>**0.05 (0.02-0.10)**	**15**<br>**0.03 (0.01-0.07)**	**15**<br>**0.01 (0.0003-0.03)**	**14**<br>**0.82 (0.22-1.33)**

Trifoilum repens							
Bosnia and Herzegovina, Spreča river valley, Donja Lohinja, flooding area, March 2020, above-ground plant parts DW, native plants, Murtić et al. [40]	3<br>0.22	3<br>0.58			3<br>0.228	3<br>0.16	3<br>0.73
France, closed smelter, autumn 2004, spring and autumn 2005, above-ground parts, DW, sown plants, Bidar et al. [42]	3<br>0.34 (0.24-0.54)					3<br>0.04 (0.019-0.068)	3<br>0.12 (0.07-0.14)
**Summarised**	**6**<br>**0.28**	**3**<br>**0.58**	**-**	**-**	**3**<br>**0.228**	**6**<br>**0.1**	**6**<br>**0.43**
**Varaždin, Croatia, this study**	**15**<br>**0.07 (0.01-0.18)**	**15**<br>**0.44 (0.13-0.71)**	**15**<br>**0.005 (0.003-0.008)**	**15**<br>**0.05 (0.02-0.08)**	**15**<br>**0.02 (0.001-0.06)**	**15**<br>**0.006 (0.001-0.015)**	**14**<br>**0.52 (0.08-1.63)**

N – number of samples, DW – dry weight

Table S11. Mann-Whitney U test with examined differences in heavy metal content between washed and unwashed plant samples of *Taraxacum officinale*, *Plantago lanceolata*, and *Trifolium repens*. Values marked in red are significant at *p* <0.050.

	**Z**	***p*-level**	**Valid N**	**Valid N**	**2*1sided**
Taraxacum officinale					
Cd	0.02	0.98	16	16	0.99
Cu	0.60	0.55	16	16	0.56
**Fe**	**2.49**	**0.01**	**16**	**16**	**0.01**
Ni	-0.32	0.75	16	15	0.77
Mn	-1.26	0.21	16	16	0.21
**Pb**	**3.51**	**0.0004**	**14**	**16**	**0.0002**
**Zn**	**2.71**	**0.01**	**16**	**16**	**0.01**
Plantago lanceolata					
**Cd**	0.08	0.93	15	15	0.93
Cu	3.10	0.002	15	14	0.001
**Fe**	**2.38**	**0.02**	**15**	**15**	**0.02**
**Ni**	-0.19	0.85	15	15	0.87
Mn	2.78	0.01	15	15	0.004
**Pb**	**2.32**	**0.02**	**15**	**15**	**0.02**
**Zn**	**-0.92**	**0.36**	**15**	**14**	**0.38**
Trifloium repens					
**Cd**	**3.70**	**0.0002**	**15**	**15**	**0.0001**
**Cu**	**4.09**	**0.00004**	**15**	**15**	**0.00001**
**Fe**	**4.13**	**0.00004**	**15**	**15**	**0.00001**
**Ni**	1.35	0.18	15	15	0.19
**Mn**	**4.17**	**0.00003**	**15**	**15**	**0.000004**
**Pb**	**2.63**	**0.01**	**15**	**15**	**0.01**
**Zn**	-0.13	0.90	15	14	0.91

N – number of samples.

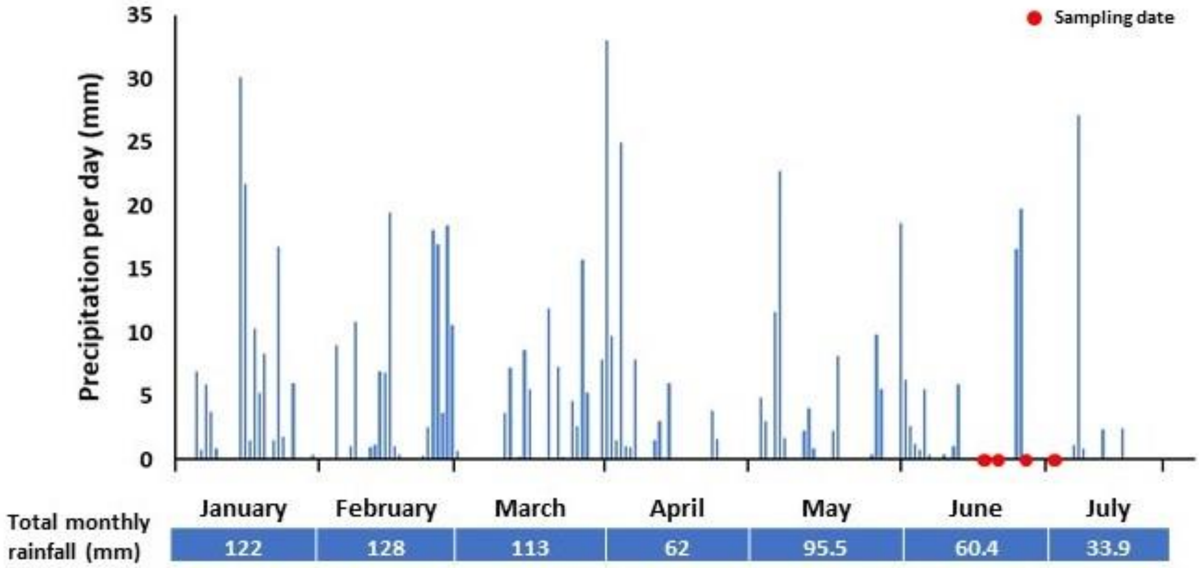

Figure S1. Total monthly precipitation and precipitation per day (mm) for the period from January to July 2013. Source of data: Meteorological and Hydrological Service of Croatia, Zagreb [90].

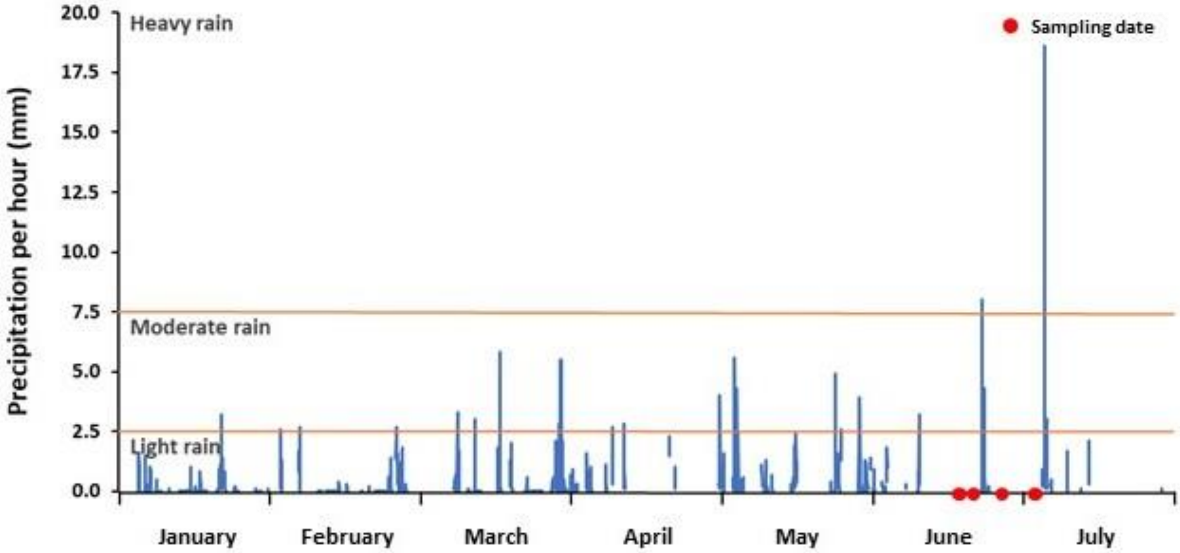

Figure S2. Precipitation amount per hour (mm) and intensity range for the period from January to July 2013. Source of data: Meteorological and Hydrological Service of Croatia, Zagreb [90].
